# Direct Ink Writing of 3D‐Structured All‐Carbon Electrodes with High Electrical Conductivity for (Vanadium) Redox Flow Batteries

**DOI:** 10.1002/advs.202417641

**Published:** 2025-05-15

**Authors:** Pablo Rodríguez Lagar, Alejandro Concheso, Daniel Barreda, Zoraida González, Miguel A. Montes‐Morán, J. Angel Menéndez, Clara Blanco, Ricardo Santamaría, Victoria G. Rocha

**Affiliations:** ^1^ Instituto de Ciencia y Tecnología del Carbono INCAR‐CSIC C/Francisco Pintado Fe, 26 Oviedo 33011 Spain

**Keywords:** carbon‐based electrodes, coal tar pitch, direct ink writing, redox flow batteries

## Abstract

Redox flow batteries are attractive systems for large‐scale energy storage due to their capability to uncouple energy and power but still need to make several improvements to reach full commercial scale. The need to search for better components, including electrode materials that allow the internal flow of electrolytes and have optimal electrochemical performance is a hot topic in the development of this kind of battery. The use of direct ink writing technology to engineer complex electrode materials both in the architecture and chemical composition opens a new field of research to optimize electrode performance. In this study, several formulations are prepared using graphite, multiwall carbon nanotubes, and two different Polyacrylonitrile (PAN)‐based short carbon fibers. Furthermore, a graphitizable binder is added to the formulation to help consolidate the printed object into a highly conductive (3000–8000 Sm^−1^) and mechanically resistant carbon electrode after a moderate heat treatment (800 °C). The 3D electrodes are successfully tested in an all vanadium redox flow cell showing a competitive performance when compared to benchmark electrodes (graphite felts).

## Graphical abstract

The study pioneers highly conductive and mechanically robust all‐carbon electrodes using Direct Ink Writing and optimizes their chemical composition for electrochemical performance. The article describes the design of a custom redox flow battery cell to test 3D printed electrodes and discusses their potential use for vanadium redox flow batteries in comparison with a benchmark material.

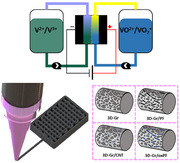

## 1 Introduction

Redox flow batteries (RFBs) are suitable devices for large‐scale energy storage thus considered one of the most realistic solutions for facilitating the cost‐effective utilization of renewable energy.^[^ ^1^ ^]^ Among them, vanadium redox flow batteries (VRFBs) are the most developed ones to date, although there are still several aspects that need to be addressed in order to further develop this technology making it more efficient and reliable.^[^ ^2^, ^3^ ^]^ Research on improving RFBs systems' key components^[^ ^4^ ^]^ include new electrolyte chemistries,^[^ ^5^, ^6^, ^7^, ^8^, ^9^ ^]^ new and more specific and efficient membranes,^[^ ^7^ ^]^ and new electrodes. Focusing on the latter, the most widely used electrodes for this application are carbon or graphite felts, cloths, and papers (GFs) thanks to their low cost, excellent mechanical and electrical properties, and corrosion resistance.^[^ ^8^ ^]^ However, GFs still have shortcomings including uncontrolled composition, fixed geometry and limited electrochemical activity toward the vanadium redox reactions.^[^ ^10^ ^]^ In addition, very few companies worldwide produce these fibers,^[^ ^11^ ^]^ and their supply could be compromised. Therefore, innovative approaches for enhancing electrode structure and composition are requested to achieve high‐performance VRFBs.

Research on VRFBs electrodes has not only been limited to electrode composition but also to electrode structure/arrangement. This latter aspect is related to some of the issues that have been identified as critical to improving the performance of the VRFBs, i.e., transport‐related issues.^[^ ^12^, ^13^ ^]^ Specifically, electrode design impacts the transport of active species, ions, and electrons.^[^ ^12^ ^]^ Innovative approaches for enhancing electrode structure include the fine‐tuning of catalysts to decouple the activation and mass transport processes, by placing atomic catalysts on scaffolds with tightly controlled pore sizes to improve the diffusion of active species to active sites.^[^ ^14^, ^15^ ^]^ Other approaches to obtain electrodes with desired designs rely on additive manufacturing (AM) technologies,^[^ ^16^, ^17^ ^]^ which had already demonstrated success in flow frames fabrication,^[^ ^18^ ^]^ in prototyping novel flow reactors,^[^ ^19^ ^]^ and the 3D printing of complete RFBs.^[^ ^20^, ^21^, ^22^ ^]^ The use of AM for RFBs electrode fabrication has focused on improving the mass transport while maintaining acceptable active surface areas and pressure drop. Thus, researchers used an indirect 3D printing approach to fabricate 3D structured metallic (Ti, Ni, or stainless steel) electrodes with geometries based on static mixers, which allowed to minimize dead zones and to control the flow profile leading to a reduction of the pumping power by two to three orders of magnitude.^[^ ^23^, ^24^, ^25^, ^26^, ^27^ ^]^ Similarly, 3D stainless steel electrodes printed using selective laser sintering (SLS) techniques showed mass transport coefficients similar to those of reticulated vitreous carbon with 60 pores per inch.^[^ ^20^ ^]^

The use of metals could compromise the chemical stability of the electrodes in heavily alkaline or acidic media, and some of the just mentioned works minimized electrode corrosion by spraying graphite particles on their surface,^[^ ^23^, ^24^, ^25^, ^26^ ^]^ or by Ni electrodeposition.^[^ ^20^ ^]^ A few works have already explored the possibility of manufacturing 3D‐printed all‐carbon electrodes for RFBs. Thus, stereolithography (SLA) has been used to obtain intricate structures of photocurable resins that were subsequently carbonized, rendering all‐carbon electrodes that reduced the pressure drop while enhancing mass transfer in RFBs.^[^ ^17^, ^28^, ^29^ ^]^ Direct ink writing (DIW) has been also used to manufacture these electrodes, specifically graphene aerogels,^[^ ^30^, ^31^, ^32^, ^33^ ^]^ with improved mass transfer in flow reactors. All these carbon electrodes showed either limited mechanical properties or electrical conductivities (or both).

The prospect of DIW electrodes from carbon‐based pastes has spurred the present study, aiming at the preparation of highly conductive and mechanical‐resistant materials. DIW^[^ ^34^, ^35^, ^36^ ^]^ is possibly the most versatile AM technology in terms of materials development, as it enables the creation of complex 3D shapes using any material or materials combination by formulating a paste with controlled rheology (a shear‐thinning yield stress fluid). The key aspect here is to prepare pastes with high loads of highly conductive carbon materials (e.g., graphite). Furthermore, an optimum paste formulation strategy should allow the integration of different carbon ingredients to improve the mechanical properties and, overall, the electrochemical response of the printed electrodes.

In addition to the abovementioned works on RFBs electrodes, various studies have already shown the limitations of DIW when preparing carbon electrodes for different energy storage devices. In that sense, the most straightforward route has been to work with carbon paste formulations based on organic solvents, binders, and additives that are not removed from the final 3D object,^[^ ^37^, ^38^, ^39^, ^40^, ^41^ ^]^ which normally brings about electrodes lacking suitable mechanical properties. This latter inconvenience was partly overcome, within the same non‐sacrificial binder approach, by developing dense DIW electrodes based on epoxy or thermoplastic polymers,^[^ ^42^, ^43^, ^44^, ^45^, ^46^ ^]^ but the electrical conductivity of those 3D structures is ≤ 10^2^ Sm^−1^. Similar materials were also developed by Pumera´s group by 3D printing of graphene/PLA composites using a different AM technology (fused filament fabrication rather than DIW).^[^ ^47^, ^48^ ^]^

The use of carbon materials pastes containing carbonisable binders is a well‐established molding technology to obtain 3D‐structured carbons, from pellets^[^ ^49^ ^]^ to carbon monoliths.^[^ ^50^ ^]^ Thus, a carbon precursor (i.e., a substance with a significant carbonization yield) is used to bind carbon particles in a “green” 3D carbon structure that is lately carbonized to render an all‐carbon material. This concept has been partially transferred to the DIW of graphene aerogel electrodes for RFBs.^[^ ^31^ ^]^ Furthermore, a similar approach was adopted to obtain aqueous colloidal carbon pastes with high (> 40 wt.%) solids loading for non‐electrochemical applications. For instance, Ajayan et al.^[^ ^51^ ^]^ developed pastes based on graphite and a mixture of carboxymethyl cellulose (CMC) and nanoclay. Miranda et al.^[^ ^52^ ^]^ also prepared graphite/CMC pastes with high graphite contents (> 50 wt.%).

DIW of graphite‐based aqueous pastes is not a simple task due to rheological and paste consolidation issues. Thus, the low hydrophilic nature of most non‐oxidized carbon materials make the aqueous pastes very difficult to homogenize and promotes particle segregation as the printing stresses build up in the DIW syringe. However, very recently García–Tuñon et al.^[^ ^53^ ^]^ combined 25 wt.% PluronicF127 in water to load graphite powders and formulate a set of printable pastes (up to 50 wt.% in graphite) by DIW. A key component in this DIW paste formulation seems to be the 25 wt.% PluronicF127 in water. Pioneered by ceramists in DIW,^[^ ^54^ ^]^ this thermo‐responsive hydrogel has increasingly demonstrated as the best carrier for the formulation of DIW pastes containing materials of a very diverse nature.^[^ ^55^, ^56^, ^57^, ^58^ ^]^ In addition, using 25 wt.% PluronicF127 in water for printing carbon‐based pastes also brings the advantage of leaving no residue^[^ ^56^ ^]^ after consolidation/carbonization, thus showing great potential for electrochemical applications.

In this study, novel carbon‐based pastes for DIW have been developed to enable electrodes for VRFB. The pastes were water‐based formulated with graphite powder, multiwall carbon nanotubes, and/or short carbon fibers as electrode active materials, and a graphitisable binder to enable a varied composition, highly structured and highly conductive electrodes. The as‐DIW electrodes were consolidated in an inert atmosphere at high temperature, being the role of the graphitisable binder critical to ensure mechanical stability and excellent electrical properties. The 3D grid‐like electrodes were tested as positive and negative electrodes toward the vanadium redox processes in three‐electrode cells, showing promising results and remarkable differences depending on the composition. Finally, the best‐performing electrodes were validated in a custom‐made VRFB.

## 2 Carbon Electrodes for Redox Flow Batteries by Direct Ink Writing

### 2.1 Pastes Formulation

Four different formulations were prepared with the aim of developing 3D electrodes for VRFB by DIW (**Table** **1**). Different carbon components were used in their composition namely graphite (Gr), multiwall carbon nanotubes (CNT), PAN‐based short carbon fibers (PF), and oxidized polyacrylonitrile (PAN)‐based short carbon fibers (oxPF). A graphitizable carbon precursor (GB) was used as a binder. A very relevant aspect that should be highlighted is that GB is also a carbon component of the pastes, i.e., it is a particulate ingredient that contributes to the total solid content of the formulation. GB powder does not dissolve in a water‐based stock solution of 25 wt.% Pluronic F127 (DI/F127) and remain unaltered in the printed electrodes. GB particles in the paste will melt and bind the rest of the carbon components in the post‐processing step as discussed in the next section. This is a significant novelty in terms of carbon‐based paste formulation for DIW since only gel‐ or resin‐like binders have been used so far.^[^ ^46^ ^]^ Graphite powder was the major component of all pastes, and it was partially replaced either by PF, oxPF, or CNT with the aim of varying the electrochemical response of the electrodes. All pastes have the same binder (GB) and paste carrier (DI/F127) weight percentages, only differing in the wt.% of the other solids (Table 1). Table S1 (Supporting Information) shows the elemental composition and the wide range of dimensions (from micron to nanometric sizes) of each raw material of the pastes. Table S1 (Supporting Information) also includes the specifications of an oxidized commercial graphite felt (oxGF) used as a benchmark for comparative purposes.

**Table 1:** Formulation of water‐based pastes for DIW.

Components [wt.%]	Gr(GB)	Gr(GB)/CNT	Gr(GB)/PF	Gr(GB)/oxPF
DI/F127 solution	49	49	49	49
GB	11	11	11	11
Graphite	40	38	30	30
CNT	–	2	–	–
PF	–	–	10	
oxPF	–	–	–	10
Total solids loading	51	51	51	51

**Figure 1:**
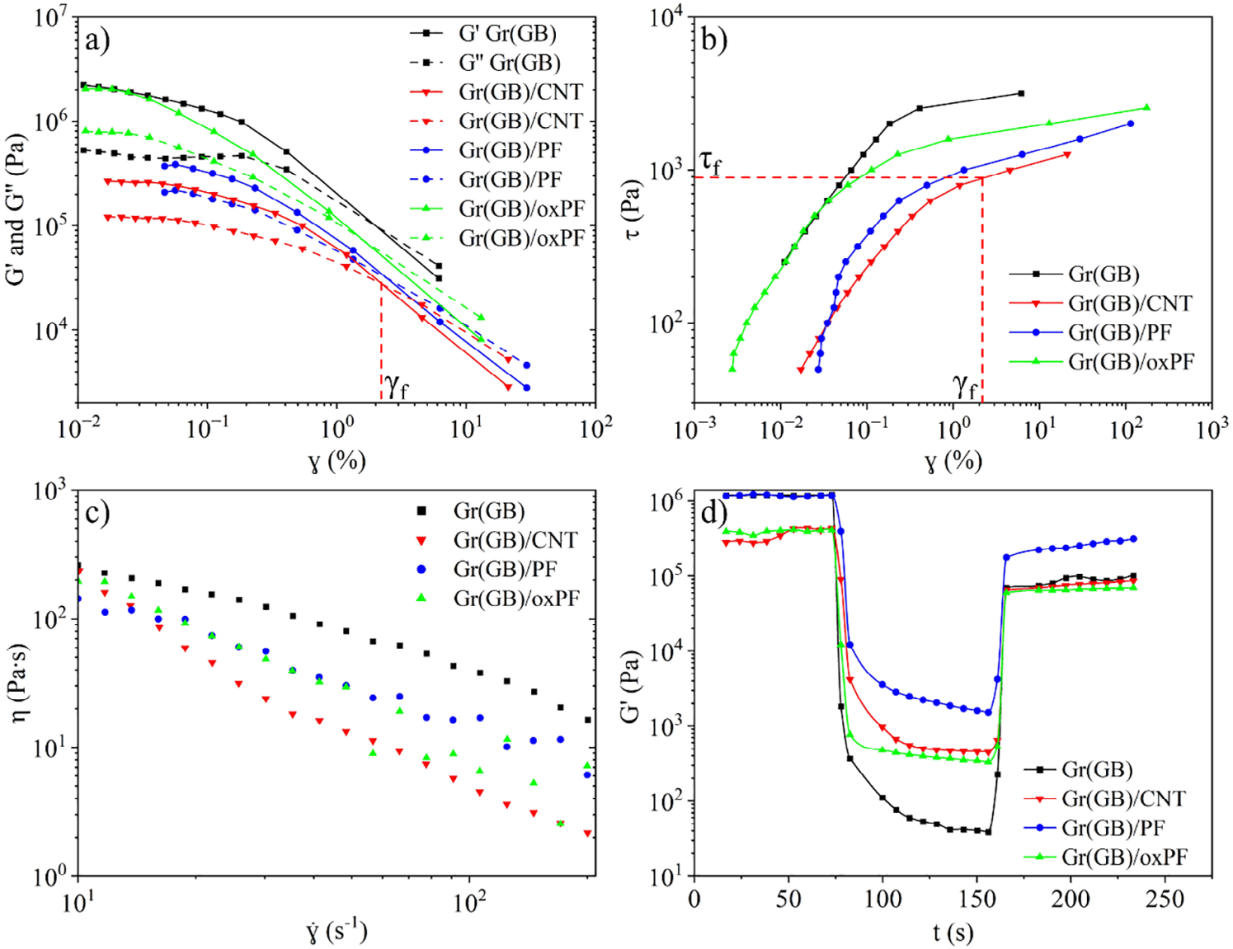
a) Amplitude sweep test (1 Hz) for the different pastes; b) shear stress from the amplitude sweep test (1 Hz); shear stress values (τ) correspond to each strain value (γ) of Figure 1a; as an example, the value of τ_f_ of the Gr(GB)/CNT paste is pinpointed from the γ_f_ value defined in Figure 1a; c) viscosity of the pastes as determined from a flow ramp test; d) three interval thixotropy test (3ITT) of the pastes.

The rheology of the pastes was initially studied. A typical graph of shear‐thinning pastes is shown in Figure S1a (Supporting Information). As a benchmark guide, the flow stress (τ_f_) determined from the strain at the crossover point (G´ = G´´) from an amplitude sweep test must be high enough (≈500 Pa) for the formulation to be self‐supportive in the absence of stress, but not too high (<3000 Pa) as this could easily compromise the extrusion process by clogging the nozzles or leading to filter press effects for filled systems. The viscoelastic properties of the formulated pastes were then determined using an oscillatory amplitude sweep test (**Figure** **1**a,b). The linear viscoelastic region (LVR) is present for all samples (Figure 1a), with equilibrium storage moduli (G’_LVR_) ranging from 2·10^5^ Pa (Gr(GB)/CNT and Gr(GB)/PF) to well above 10^6^ Pa (Gr(GB) and Gr(GB)/oxPF). These G´_LVR_ values (≥ 10^5^ Pa) guarantee a good printing resolution of multi‐material structures. After the linear viscoelastic region (Figure 1a), there is a sharp drop in the storage modulus (G’) at shear stress (τ_y_, Figure 1b) when the DI/F127 hydrogel network starts to break down, leading to the irreversible deformation of the paste. As the structure continues to break and rearrange internally, it reaches a predominantly liquid behavior at a flow stress (τ_f_, Figure 1b) ranging from 900 to 3000 Pa, depending on the type of material. As just mentioned, these τ_f_ values guarantee that all pastes are self‐supportive in the absence of stress.^[^ ^59^ ^]^

Flow ramp tests were also performed to determine the flow behavior of the pastes (Figure 1c). Due to the high solids loading, and with the aid of the paste carrier (DI/F127), the four pastes exhibit a non‐linear plastic fluid behavior, showing viscosity values above 10^2^ Pa·s at 10 s^−1^, which is ideal for DIW. Partially substituting graphite with smaller particles such as chopped short PAN fibers, oxidized chopped PAN fibers, and MWCNTs resulted in a reduction in paste viscosity. This could facilitate extrusion through narrower nozzles. The viscosity profiles and values of the pastes are very similar to that of the paste carrier, i.e., DI/F127 only,^[^ ^55^ ^]^ which suggests that it is the carrier that controls the viscosity of the carbon‐pastes.

The transition rate of the pastes from a liquid‐like to a solid‐like behavior was studied using the three‐interval thixotropy test (3ITT) performed in oscillatory mode (Figure 1d). This test mimics the flow conditions experienced by the material as it passes through the nozzle. Figure S1b (Supporting Information) illustrates the stress profile experienced by the material during the DIW process, where the maximum stress profile is reached at the nozzle tip. Ideally, to achieve high‐resolution structures, the material should immediately recover its full rest stiffness (G’_LVR_) after passing through the nozzle. However, in practice, this is rarely the case. In our case, all pastes did recover at least 80% of the initial G´_LVR_, with a minimum G’_LVR_ of 7·10^4^ Pa being achieved post‐flow (Figure 1d). This is also indicative of good levels of self‐supportive conditions. On the other hand, the G’ values of the pastes in the flow region (i.e., when G’’ > G’) exhibited time dependence, slightly decreasing as the shear stress duration increases (central part of Figure 1d). This non‐constant G’ values in the flow region are indicative of a thixotropic behavior of the paste, with the viscosity of the fluid changing (decreasing) with time. Although this could be a drawback in printing processes where maintaining a constant flow rate of the paste is crucial, this was not the case, at least in the printing conditions used in this work. Certainly, pastes are subjected to high shear stresses in the nozzle tip, but if the printing speed is not too low, those high shear stresses are only endured for short times (typically < 10 s).

### 2.2 Direct Ink Writing and Postprocessing

**Figure 2:**
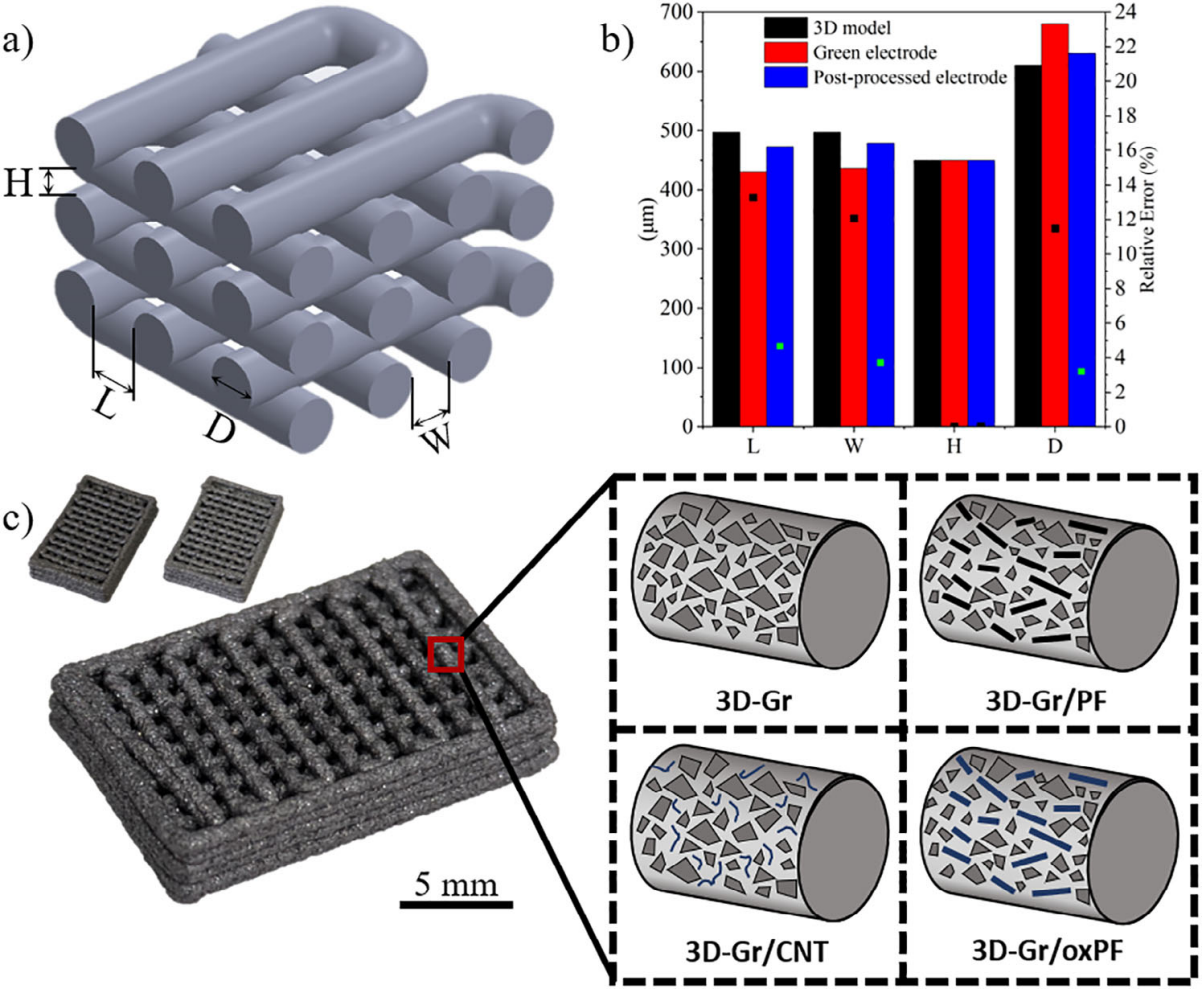
a) Grid‐like unit cell model; b) L, W, H and D sizes (Figure 2a) of the model, as‐printed and consolidated (post‐processed) electrodes; c) image of a 3D‐Gr consolidated electrode; the top‐left inset shows an electrode before (i.e., as‐printed, left) and after (i.e., consolidated, right) postprocessing; the shrinkage is negligible; d) scheme of the consolidated electrodes with different compositions.

CAD grid‐like 3D objects as shown in **Figure** **2**a were successfully printed through 610 µm nozzles with the four pastes already described. This grid‐like design was chosen as a proof of concept, with the aim of reducing the pressure drop in the battery by facilitating electrolyte flow through channels oriented in the flow direction, compared to benchmark oxidized graphite felt (oxGF in Figure S2, Supporting Information). The use of these grid‐like electrodes has already demonstrated a significant enhancement of the mass transfer in RFBs.^[^ ^31^ ^]^ The grid‐like structure consists of parallelepipeds (15×10×2.7 mm in size) of six filament layers and was designed with a 50% infill. Four additional dimensions define the structure, namely the distance between filaments in the longitudinal and transversal directions (L and W, respectively), layer height (H), and filament diameter (D) (Figure 2a,b). DIW at 10 mm s^−1^ was successfully achieved for all pastes. Printed electrodes were ambient dried for 24 h, and the printing precision as determined by the final dimensions of those as‐printed electrodes was reasonably good, with deviations below 15% of the nominal (model) values (Figure 2b). Although the as‐printed and dried electrodes were easy to handle, they did not reach the mechanical and electrical properties to operate in a VRFB.

A postprocessing step of heat treatment under an inert atmosphere was then required to improve the properties of the printed (and dried) electrodes. This heat treatment has two effects on the printed electrodes, i) the paste carrier (DI/F127) decomposes; and ii) the GB particles melt and bind the other carbon particles (graphite, CNTs, and PFs when present) in the paste together. The timing of those two effects is crucial, i.e., melting of the GB particles is required to start at lower temperatures than the decomposition of the DI/F127 paste carrier. Otherwise, the electrode would crumble during the heat treatment. TG analysis of the paste carrier (Figure S3a, Supporting Information) defines the temperature window for GB (or other particulate binder) to melt. F127 starts to decompose at 300 °C and presents a maximum of its DTG curve at ≈400 °C. GB, on the other hand, first melts at 110 °C (softening point), thus ensuring the wrapping and binding of the graphite particles (or any other carbon material present in the composition) while F127 remains unaltered. As the heat treatment proceeds beyond the softening point, GB particles carbonize transforming into a carbon material that consolidates the electrodes and preserves the original shape of the 3D‐printed carbon pastes. The carbonization of GB involves the release of volatiles up to 600 °C with a maximum weight loss occurring at 370 °C (Figure S3c, Supporting Information). Both the decomposition of F127 and the volatile release of GB overlap in the TG profile of the printed electrode (Figure S3d, Supporting Information). Finally, and as expected, no significant effect of the heat treatment was found on the rest of the carbon components in terms of weight loss (Figure S3b, Supporting Information).

The postprocessing brings about tougher structures with very similar dimensions to the printed electrodes (Figure 2b). There is a slight decrease in the filament diameter (D), which in turn increases the interfilament spaces (L and W, Figure 2b). Overall, the shrinkage of the electrode after postprocessing is negligible (inset in Figure 2c). It should be clear that GB and DI/F127 are the only components of the original pastes that suffer significant volume changes during the postprocessing of the as‐printed electrodes. This fact and the relatively low amount of GB present on the initial pastes (11 wt.%, Table 1) determine the low shrinkage of the printed electrodes. Again, the thermal stability of the paste carrier is critical to buffer the volume changes caused by the GB particle's expansion (melting) and contraction (carbonization) before F127 decomposes.

**Figure 3:**
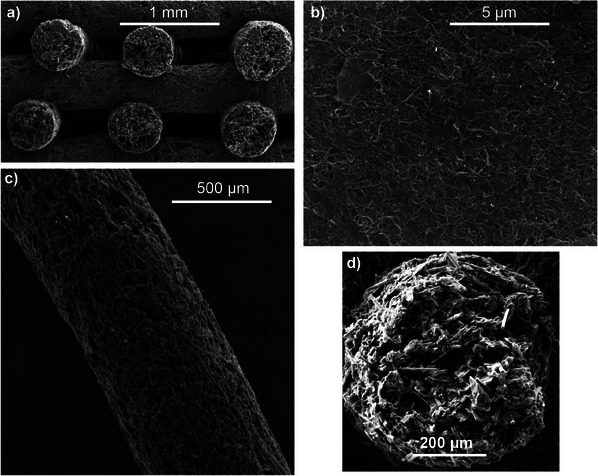
Selected SEM images of different 3D electrodes: a) cross‐section view of a 3D‐Gr electrode with the well‐defined channels to expose the inner filament surfaces to the aqueous solutions in a VFRB; b) surface detail of a 3D‐Gr/CNT electrode where the graphite flakes and carbon nanotubes are clearly depicted; c) detail of a 3D‐Gr/PF filament where the graphite flakes and carbon fibers are mixed; most fibers are oriented along the filament length; it is also noteworthy the high connectivity of the graphite particles; d) cross‐section of a filament in a 3D‐Gr/PF electrode showing reasonable good compaction of the all‐carbon material.

The resulting all‐carbon electrodes have a new composition (**Table** **2**) as calculated from the carbonization yield of GB (37%, Figure S3c, Supporting Information) and the F127 decomposition (Figure S3a, Supporting Information). The electrodes are essentially made of graphite particles, with the GB‐derived carbon only amounting to < 10% of the total weight of the structure. As a result, the XRD of all consolidated electrodes is almost identical to that of the graphite (Gr) powders (Figure S4, Supporting Information). Raman spectroscopy of the 3D‐Gr filaments shows however a composite spectrum in which both highly ordered and disordered carbon coexist on the surface of the Gr flakes (Figure S5, Supporting Information). When compared to the Raman spectra of Gr and GB‐derived carbon it is safe to infer the GB‐derived carbon coating of most if not all the Gr particles. This is schematized in Figure 2d, where the occurrence of other components in the filaments is also represented. **Figure** **3** shows selected SEM images of the consolidated electrodes to further illustrate their microscopic structure.

**Table 2:** Composition of the 3D printed electrodes after postprocessing at 800 °C estimated from TGA analysis (Figure S3, Supporting Information).

Components [wt.%]					
	Gr	GB‐derived carbon	CNT	PF	oxPF
3D‐Gr	90.6	9.4	–	–	–
3D‐Gr/CNT	86.1	9.4	4.6	–	–
3D‐Gr/PF	79.3	9.4	–	11.4	–
3D‐Gr/oxPF	79.3	9.4	–	–	11.4

**Figure 4:**
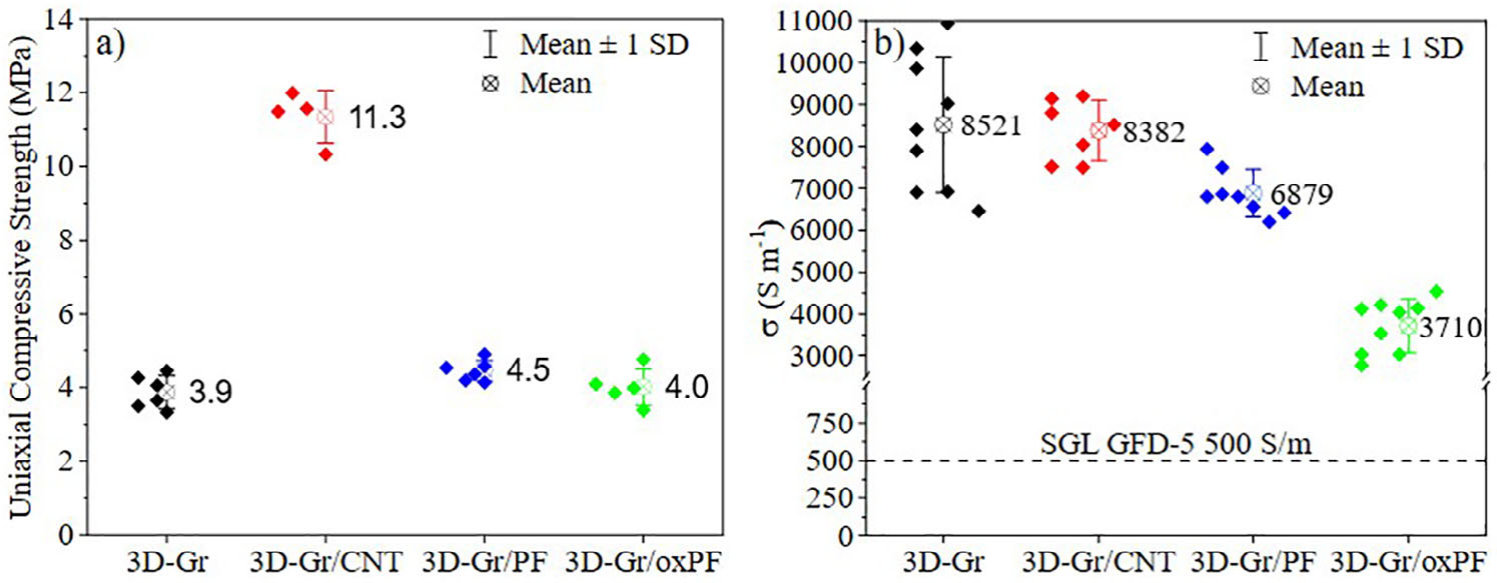
a) Uniaxial compression strength of the DIW 3D electrodes b) Electrical conductivity of the carbonized 3D carbon electrodes and a Graphite Felt (PAN‐based GF SD5 from SGL).^[^ ^63^ ^]^

The mechanical properties of the all‐carbon DIW 3D electrodes were tested under compression (**Figure** **4**a). The results show that the 3D‐Gr/CNT composition exhibits the highest uniaxial compression resistance (12 MPa), nearly three times greater than the strength of the 3D‐Gr base composition electrode. On the other hand, electrodes incorporating short PAN fibers showed no improvement over the 3D‐Gr electrodes, though they also did not experience any disruption. These values are well above the compression strength of graphene aerogels (hundreds of kPa) and are comparable to those of vitreous carbon microlattices.^[^ ^60^ ^]^

The graphite high content of the all‐carbon 3D electrodes is responsible for their outstanding electrical conductivities (Figure 4b). The worst performing electrode (3D‐Gr/oxPF) shows conductivity values of 3.7 10^3^ Sm^−1^ (Figure 4b), which is two orders of magnitude higher than that of the as‐printed and dried filaments and greater than other DIWed electrodes published (**Table** **3**). The best electrical conductivities corresponded to the 3D‐Gr and 3D‐Gr/CNT electrodes, with values of ≈8.5 10^3^ Sm^−1^ (Figure 4b). This value is not too far from the electrical conductivity of the graphite particles as determined in a four‐probe point test^[^ ^61^ ^]^ (Figure S6, Supporting Information). This suggests that the Gr volume fraction in the all‐carbon filaments is very close to the so‐called percolation threshold, i.e., the graphite flakes form a connected path within the filament.^[^ ^62^ ^]^ A more realistic physical description of the conducting network is given by effective media theories,^[^ ^63^ ^]^ assuming an intermediate idealized medium in which a good proportion of graphite flakes will contact each other but most of them are coated with much less conductive GB‐derived carbon, as discussed above in light of the Raman measurements (Figure S5, Supporting Information). This means that the conductivity of that GB‐derived carbon still influences the electrical performance of the electrodes. In that sense, the 3D objects with short PAN fibers in their formulation would also contribute to lowering the electrical conductivity values of the electrodes. This is particularly relevant in the case of the 3D‐Gr/oxPF in which oxidized short PAN carbon fibers were used in the formulation. A possible explanation for the drop observed in the electrical conductivity of this particular electrode could be related to the lower wettability of the oxidized fibers by the GB during the postprocessing that would end up in a less homogeneous distribution of the binder during melting reducing notably the conduction pathways within the filaments.

**Table 3:** Summary of the electrical conductivities of the state‐of‐the‐art DIWed all‐carbon electrodes compared with others.

Formulation	AM technology	Postprocessing	Electrical Conductivity [Sm^−1^]	Refs.
40% Gr+11% GB+Pluronic	DIW	800 °C N_2_	8500	This Work
91% Gr+9% Cellulose	DIW	400 °C N_2_	2400	[64]
42% Gr+0.65%GO+0.5% CNT+Pluronic	DIW	800 °C 90%Ar/10%H_2_	1500	[65]
40% Gr+2% Nanoclay	DIW	Ambient drying	1000	[51]
4% GO+5% Cellulose+2.5% CF+Water	DIW	1050 °C N_2_	220	[30]
2% GO+2% CaCl_2_+CB/CNT/CNF+Water	DIW	1050 °C N_2_	136	[32, 33]
25% Gr+10% CMC	DIW	Ambient drying	130	[46]
3% vol GO+Water	DIW	900 °C 90%Ar/10%H_2_	100	[66]
Photocurable resin	Stereolithography	850 °C	85	[28]
20%GO+Epoxy	DIW	Curing 220 °C	1.5	[67]
8.5% CF+2% CNT+Epoxy	DIW	Curing 220 °C	1	[44]
79.4% Ti+9% Epoxy+11.6% Glycerol	Mold Printing	1000 °C Ar + graphite spray coating	0.1	[68]

### 2.3 Electrochemical Characterization of the 3D Carbon‐Based Electrodes from DIW

**Figure 5:**
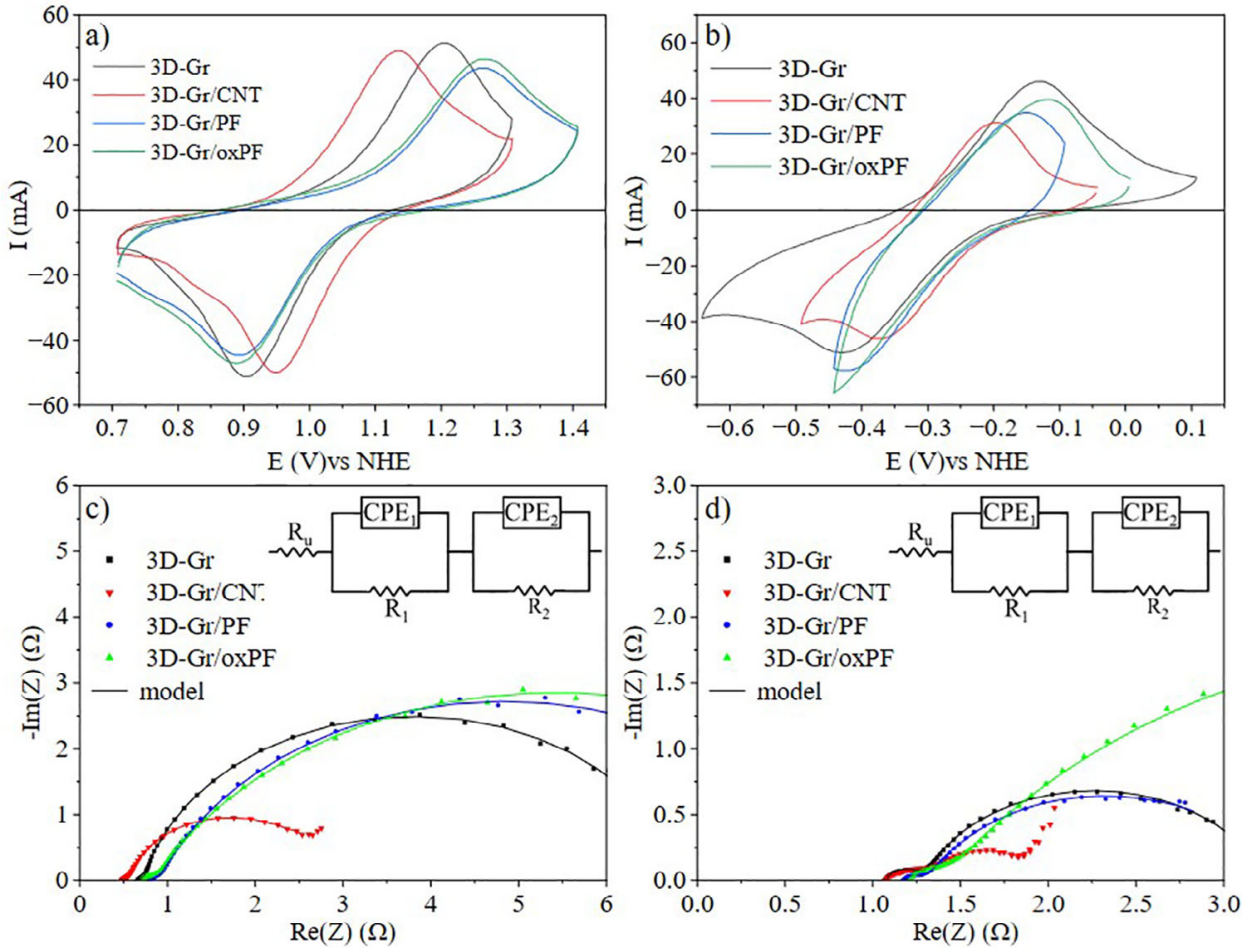
Electrochemical characterization of the 3D carbon‐based electrodes from DIW, using a three‐electrode cell and a 0.05 m VOSO_4_/1.0 m H_2_SO_4_ solution as electrolyte. a) CVs recorded at 20 mV s^−1^ on the different electrodes in the positive potential window; b) CVs recorded at the same *v_scan_*, on the different electrodes in the negative potential window; c) and d) Nyquist plots recorded on the mentioned electrodes at a frequency range of 10^6^–10^−1^ Hz.

**Figure** **5** summarizes the preliminary electrochemical characterization of the DIW electrodes under evaluation by means of Cyclic Voltammetry (CV) and Electrochemical Impedance Spectroscopy (EIS) experiments carried out in a three‐electrode cell configuration. First, Figure 5a,b show the Cyclic Voltammograms (CVs) recorded, at 20 mV s^−1^, on the different samples acting as positive and negative electrodes, respectively, using a 0.05 m VOSO_4_/1.0 m H_2_SO_4_ solution as electrolyte. According to the results, remarkable differences can be observed depending on the selected electrode in both cases. Surprisingly, the worst behavior was observed when using those electrodes having short carbon fibers (3D‐Gr/PF and 3D‐Gr/oxPF) in their composition. A higher overpotential toward the VO^2+^ oxidation and a wider peak potential separation was observed on these electrodes when compared to 3D‐Gr, which could be indicative of their higher charge transfer resistance (Figure 5a and Table S2, Supporting Information) However, the incorporation of CNTs to the electrode composition (3D‐Gr/CNT) led to an improved behavior toward the same faradaic process, showing a much lower anodic overpotential. These better results could be explained by considering the high electrical conductivity of CNTs distributed on the electrode´s surface (Figure 3b) and their potential to increase the active surface area.^[^ ^69^, ^70^ ^]^ Despite the excellent performance in the positive half‐cell, the presence of CNTs on 3D‐Gr/CNT acting as negative electrode (Figure 5b) impacts negatively the electrochemical performance of the corresponding half‐cell as not only the processes ascribed to the V^3+/2+^ redox pair are boosted but also the undesired hydrogen evolution reaction (HER), with could decrease the coulombic efficiency of the whole cell. Looking for a compromise allowing to achieve a suitable device in terms of columbic, voltage, and energy efficiencies, the 3D‐Gr grid‐like electrode is postulated as a suitable negative electrode.

Further analysis of the behavior of the DIW electrodes in the positive half‐cell (Table S2, Supporting Information) shows that currents measured (both anodic and cathodic) are rather similar on the four electrodes under evaluation being slightly higher for 3D‐Gr. More significant is that the *Ia/Ic* ratio is very close to 1 for all of them indicating that oxidation and reduction reactions develop to the same extent. The highest differences are found in the ΔE, which is significantly smaller for the 3D‐Gr/CNT electrode, indicating a much higher reversibility of the redox process when CNT is added to the formulation. In the same context, Figure 5c shows the Nyquist plots recorded on the samples acting as positive electrodes, corroborating that the presence of CNTs in the 3D electrode formulation (3D‐Gr/CNT) greatly reduces the charge transfer resistance of the anodic reaction (smaller semicircle diameter) which greatly contributes to better electrochemical performance.

On the other hand, the electrochemical behavior of the same 3D electrodes in the negative half‐cell is quite different, as observed in Figure 5b and Table S2 (Supporting Information). The worst results are observed when analyzing the *Ia/Ic* ratio, as in all the four electrodes they are clearly much lower than 1, indicating that the cathodic processes (both V^3+^ reduction and HER) are more intense than the anodic one. As previously stated, this effect cannot be ascribed to an unsuitable V^3+^/V^2+^ redox reaction reversibility, but to the presence of the parasitic HER. Only the 3D‐Gr electrode provides an *Ia/Ic* ratio of 0.91, being clearly the best electrode for the negative half‐cell. Remarkably, the presence of short fibers has a very negative impact on the performance of the electrode in this half‐cell as HER predominates over those reactions related to the electroactive vanadium species.

Based on an equivalent circuit (EC) model applied to Nyquists plots similar to Schilling et al,^[^ ^71^ ^]^ three elements represent each half‐cell, matching the EIS experimental data (Figure 5c,d; Figure S7, Supporting Information): R_u_ for ion transport resistance, R_1_ for transport through the electrode, and R_2_ for charge transfer. The R_u_ and R_2_ values are significantly lower in electrodes with CNTs, confirming that CNTs in the 3D‐Gr/CNT formulation reduce charge transfer resistance, enhancing electrochemical performance. R_1_ is negligible for these electrodes. For the negative electrode (Figure 5d; Figure S7b, Supporting Information), R_u_ and R_1_ are slightly higher than for the positive electrode in all formulations, but R_2_ values are much smaller, indicating lower charge transfer resistance in the negative electrode. Again, the 3D electrode containing CNT shows smaller charge transfer resistance values, indicating that this redox process is favored on 3D‐Gr/CNT. However, the HER is now more relevant, as seen in the larger cathodic current.

Despite these experiments already demonstrating that the electrochemical performance of the 3D electrodes under study can be modified through the formulation/composition of the related carbon‐based pastes, the stationary nature of the experiments performed (both CV and EIS) could limit the evidence of real differences in their electrochemical behavior due to diffusional limitations. In this sense, despite Figure 5a showing that similar currents were measured on the different electrodes submitted to CV experiments, additional results were obtained in a flow‐through three‐electrode setup (described in Figures S8 and S9, Supporting Information) and a rotatory disk electrode (RDE) configuration with a 100% infill the 3D printed geometry required for these experiments (Figure S10, Supporting Information, and Figure S11 and Table S3, Supporting Information, for the results obtained with this set up). As discussed in the Supporting Information the results obtained for 3D‐Gr and 3D Gr/CNT evidenced much more clearly their differences in performance than those obtained in stationary experiments.

### 2.4 Custom‐Made RFB Assembly and Testing Using 3D Carbon‐Based Electrodes from DIW

**Figure 6:**
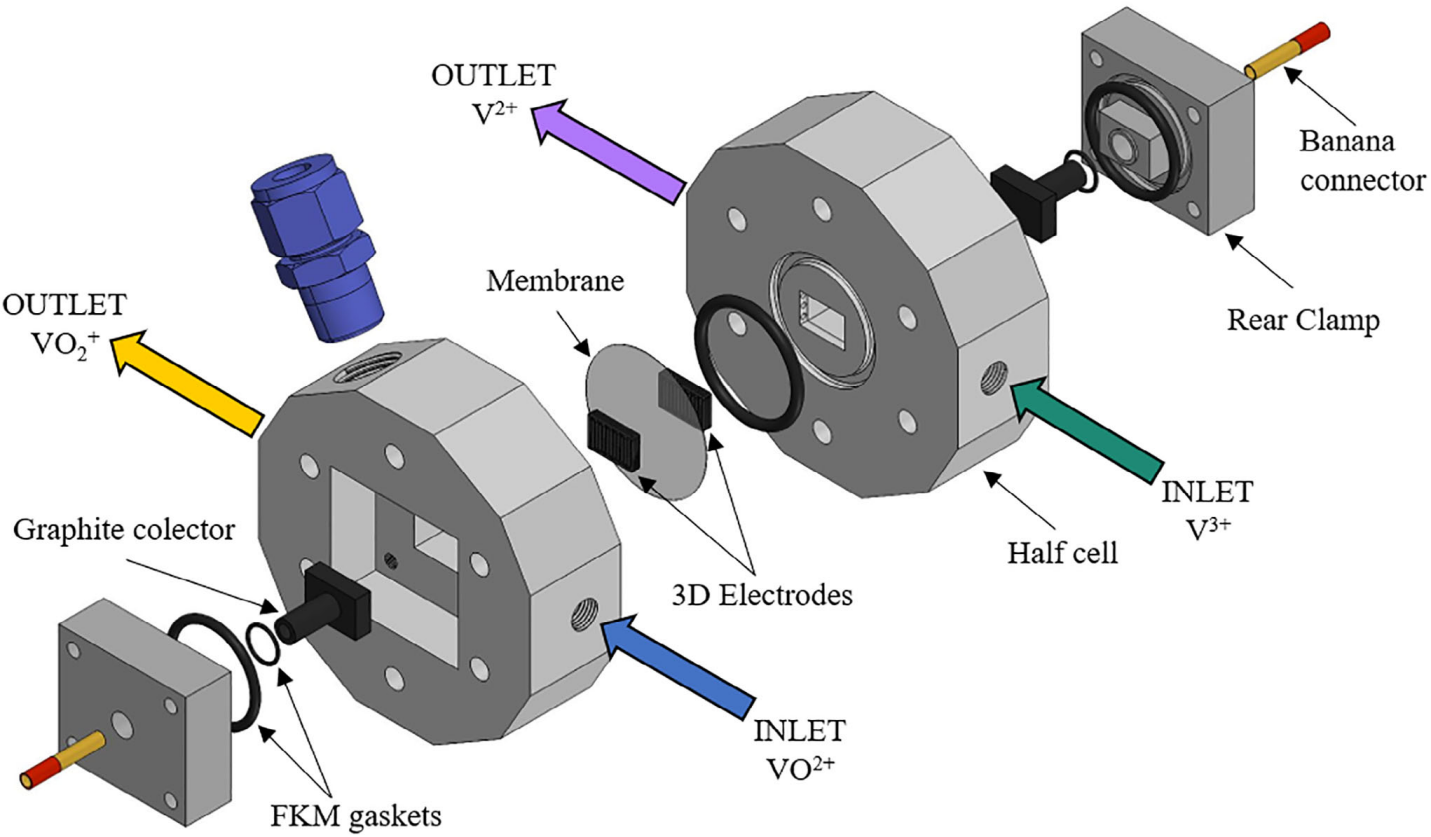
Scheme of the custom redox flow battery cell assembly using 3D carbon‐based electrodes manufactured by DIW.^[^ ^72^ ^]^

A custom‐made redox flow battery aiming to corroborate not only their appropriateness from the electrochemical point of view but also their mechanical stability under real flow conditions was built and tested. At this point, it is important to highlight that previous results found in the literature showed important drawbacks related to the mechanical stability of carbon‐based samples/objects manufactured by DIW such as aerogels^[^ ^33^, ^56^ ^]^ which were quite weak, or to their poor electrical conductivity due to the use of polymeric resins as binders.^[^ ^46^ ^]^ However, the formulations of the pastes used in this work using a graphitizable binder (GB) and the post‐processing step performed enabled the achievement of robust electrodes that could be fitted in a suitable flow cell. Therefore, a custom design of a flow‐through RFB cell to fit in the manufactured electrodes (10×15×2.7 mm^3^) was explored (**Figure** **6**). A further description as well as all the CAD drawings of the cell components are providedelsewhere.^[^ ^72^ ^]^

Before testing the electrodes in the RFB cell, pressure drop and wettability tests were performed to ensure success in the cell operation. Pressure drop tests were performed for the base composition of DIW 3D electrodes (i.e., 3D‐Gr) as the four compositions studied are geometrically identical, and also for the benchmark material labeled oxGF (commercial oxidized graphite felt, Figure S2, Supporting Information). The grid‐like geometry (3D‐Gr) showed a lower pressure drop than the benchmark felt and the differences are higher at increasing flow rates confirming the suitability of the chosen 3D geometries (Figure S12, Supporting Information). Wettability tests against the electrolyte (0.5 m VOSO_4_ and 1 m H_2_SO_4_) were performed on 5 layer electrodes, 100% infill 10×10 mm^2^ (Figure S13b, Supporting Information), for each composition confirming that the DIW 3D all‐carbon electrodes exhibit excellent behavior (Videos S1–S4, Supporting Information) as the electrolyte readily penetrates the specimens.

**Figure 7:**
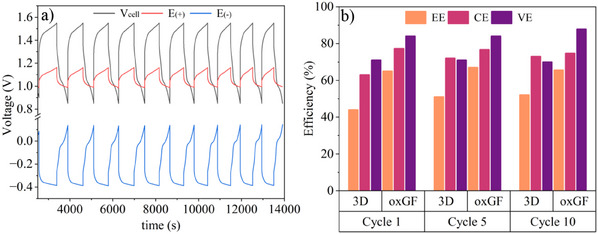
a) Galvanostatic charge/discharge tests performed (applying 15 mA cm^−2^ from 0.85 to 1.55 V operational voltage range) in the custom‐made RBF cell using 3D‐Gr/CNT and 3D‐Gr as positive and negative electrodes, respectively; b) efficiencies of the VRFB as a function of cycles for DIW 3D electrodes and for the benchmark oxidized commercial graphite felt (oxGF): EE, energy efficiency; VE, voltage efficiency; CE, culombic efficiency.

This RFB cell was then first assembled fitting in the 3D‐Gr/CNT as positive and 3D‐Gr as negative electrodes, respectively, and galvanostatic charge–discharge tests were performed (**Figure** **7**a).

Bearing in mind the results from previously discussed three‐electrode configuration experiments under static conditions, this custom‐made RFB cell allows a much easier identification of its limitations. For instance, as it was observed in the static setup, the performance of the positive electrode (3D‐Gr/CNT, red curve, Figure 7a) (displaying better reversibility) is much better than the performance of the negative electrode (3D‐Gr, blue curve, Figure 7a), thus confirming that the composition of the 3D object acting as negative electrode needs to be optimized in order to further enhance the whole cell performance. As can be seen, stable charge–discharge cycles, providing a voltage efficiency (VE) of close to 70% were achieved (Figure 7b). This performance is similar to that obtained by Li et al.^[^ ^32^ ^]^ for the so‐called “3 mm pore” graded DIW graphene/carbon black aerogels electrodes (30 mm x 30 mm x 3 mm) in a commercial flow cell.

Additionally, charge–discharge tests were also performed in the custom cell assembled with oxidized graphite felt (oxGF) as positive and negative electrodes making this material a representative benchmark material. The results in comparison with the novel DIW electrodes are shown in Figure 7b. Coulombic efficiencies (CE) are similar for both cells (≈75%), but not the voltage efficiency (VE) (and therefore the energy efficiency), as it was lower if the DIW electrodes were used (70% vs 85%). We ascribe this underperformance of the 3D‐printed electrodes to two main factors. First, the external surface area of the oxGF is much higher than that of the 3D grid‐like structure of the same dimensions. According to data shown in Figure 2b (filament diameter of 630 µm), geometrical calculations based on the model 3D electrode depicted in Figure 2a give an external surface of 10.5 cm^2^ for the all‐carbon electrodes. A simple calculation of the external surface area of the oxGF felt, considering filaments of 7 µm diameter randomly organized with a filament density of 2 g cm^−3^, renders a value of ≈180 cm^2^. Although that area is most likely overestimated (i.e., it does not account for the filaments overlapping areas, especially relevant as the felt is compressed in the VRFB cell), the difference between the two systems is still overwhelming. This can be graphically depicted by expressing the CVs of Figure 5 in terms of surface‐specific current intensities (Figure S14, Supporting Information). The second factor that contributes to the lower voltage efficiency of the 3D‐printed electrodes is the ohmic losses caused by poorer contact with the current collectors. This is a consequence of the rigidity of the printed electrodes when compared to the oxGF felts.

In addition, the cell design of Figure 6 was constrained to the 3D electrodes small size, thus resulting in a very small reaction volume which also contributes toward ohmic losses if the operation is pushed toward higher flow rates or current densities. This fact made our cell difficult to compare with commercial designs but still by comparison with the oxGF benchmark in the same RFB cell enables us to validate the DIW 3D electrodes for this application.

Still, and as a fundamental outcome of the present work, the high conductivity of the 3D‐printed electrodes is able to compensate for such important drawbacks and perform reasonably well in the custom VRFB. Moreover, there is plenty of room for improvement. In terms of electrical conductivity and mechanical properties, it is possible to increase the graphite content on the paste formulations by up to 60%. Also, a higher temperature treatment should enhance the conductivity of the electrodes by boosting the conductivity of the GB‐derived carbon. As for the increase in the external surface area of the printed electrodes, thinner filaments could be attainable using more sophisticated 3D printers. Furthermore, future works exploring more complex electrode 3D geometries (beyond the grid‐like design used in this work as a proof of concept) and more efficient formulations (including the addition of different carbon or non‐carbon ingredients to the pastes) for this specific application are expected to come in this promising area. The optimization of the surface properties of the electrodes and the operational conditions of the VRFB cells are also envisaged as relevant aspects to be explored to improve the sustainability goals of this technology.

## 3 Conclusion

DIW has been demonstrated to shape various carbon formulations in a complex structure that allows the flow of electrolytes in a custom RFB cell. A carbon precursor GB has been used successfully as a particulate binder that consolidates the as‐printed object after thermal treatment, rising a composite object with excellent mechanical and electrical properties that can be used as an electrode in an all‐vanadium redox flow battery. The best formulation to be used as a positive electrode contains GB, graphite, and CNT, being the last component essential to accelerate the redox reactions. The base composition of GB and graphite particles has been demonstrated as the best negative electrode as it minimizes hydrogen evolution.

The best electrodes have been combined in a custom‐made flow cell to fit in the DIW grid‐like electrodes and tested successfully as a fully functional RFB. The performance of the printed electrodes is competitive to that obtained with carbon/graphite felts, especially considering differences in the external surface area and electrode‐collector contact issues. Furthermore, it has been demonstrated that contrary to carbon felts, the composition can be modulated to optimize electrochemical performance. Formulations of pastes for DIW can be easily modified adding new carbon materials that increase reactivity or increase surface area, or other additives can be added to the formulations (or in the post‐processing) to inhibit undesired chemical reactions as HER. The additives are not necessarily restricted to carbons, but other materials are likely to be beneficial for electrode performance (to improve wettability for example). It is also relevant to address the research in new electrode architectures that can be printed and help to upgrade the mass transfer processes and reduce pressure losses in the device, optimizing the fluid‐dynamics properties of the electrodes.

## 4 Experimental Section

### Raw Materials

Five different carbon materials were used in the paste's formulation:

1. Synthetic graphite (Gr) (D_90_ = 56 µm) TIMREX KS75 sieved below 75 µm, supplied by TIMCAL Graphite and Carbon.
2. A graphitizable binder (GB) consisting of a coal tar pitch (typical binder used for graphite electrode manufacturing^[^ ^73^ ^]^) with a softening point (SP) = 110 °C supplied by Química del Nalón, S.A also ground and sieved below 75 µm.
3. Multiwalled carbon nanotubes (CNT) (> 90% carbon basis, 110–170 nm diameter, supplied by Merck)
4. Short PAN carbon fibers (PF) (SIGRAFIL C M80‐4/240‐UN, supplied by SGL Carbon)
5. Oxidized short carbon PAN fibers (oxPF), from a lab‐made oxidation treatment at 500 °C‐1 h, under airflow (200 mL min^−1^)
6. For comparative purposes, an oxidized graphite felt (oxGF) was also used. It was obtained after the thermal treatment of a commercially available felt (SIGRACELL GFD 4.65 EA, supplied by SGL) at 550 °C–1 h under airflow (200 mL min^−1^). It was important to remark that this carbon‐based felt had been previously reported as a suitable active electrode material for this application.

PluronicF127, supplied by Sigma–Aldrich, and deionized water (DI) were also used as raw materials for paste formulation. Specific properties of the listed raw materials are shown in Table S1 (Supporting Information).

### Pastes Formulation

Four different pastes were formulated with a final solid loading of 51 wt.%. As a starting point, a water‐based stock solution of 25 wt.% Pluronic F127 (DI/F127) was prepared and kept overnight at 4 °C up to complete dissolution of Pluronic F127. The wt.% of each component in each paste is shown in Table 1 and their labeling was as follows: Gr(GB), Gr(GB)/PF, Gr(GB)/oxPF, and Gr(GB)/CNT. The gradual addition of the appropriate amount of each component over this stock solution was aided by a Speed Mixer (SpeedMixer DAC 150.1 FVZ‐K) at 2000 rpm and 1 min cycles and its subsequent cooling for 20 min at 4 °C.

### Electrolyte Solution

A 0.05 m vanadium (IV) oxide sulfate hydrate (97% VOSO_4_·xH_2_O, supplied by Sigma–Aldrich) in 1.0 m H_2_SO_4_ (99.99 wt.%, supplied by VWR) solution was used in different electrochemical experiments carried out. For the evaluation of the custom‐made RFB cell performance, 0.5 m V^3⁺^ and VO^2⁺^ solutions were prepared for use as anolyte and catholyte, respectively. These species were produced via the electrochemical transformation of a 0.5 m VO^2+^ (97% VOSO_4_ xH_2_O, Sigma–Aldrich) in a 1.0 m H_2_SO_4_ (VWR) solution, following the procedure described elsewhere.^[^ ^74^ ^]^

### Pastes Rheology Characterization

The rheological properties of the different pastes were evaluated using a rheometer (HAAKE MARS III, ThermoFisher) at a plate‐plate geometry (20 mm diameter, 500 µm gap). The temperature was set at 25 °C for all the measurements. To avoid humidity losses during the tests the gap edges were coated with a thin layer of paraffin oil. The frequency was set as 1 Hz for all the oscillatory tests. Flow behavior was analyzed in rotational mode, aiming to replicate the shear rates achieved at the nozzle during extrusion. A range of shear rates between 0 and 200 s⁻¹ were studied. For the characterization of viscoelastic properties, amplitude sweep tests were conducted up to shear stress values of 5000 Pa. Additionally, to assess elastic recovery, Three Interval Tests (3ITT) in oscillatory mode were performed.

### Electrodes Direct Ink Writing (DIW) Methodology

The water‐based formulated pastes were filled into 3 mL syringes (Adhesive Dispensing) which were subsequently loaded into a custom‐built DIW printer modified in the group. Details of the printer were published elsewhere.^[^ ^75^ ^]^ 610 µm nozzle tips were used to print parallelepiped (10×15×2.7 mm) grid‐like designs, consisting of 6 filament layers where the layer overlap was 26% to ensure good contact between layers and a printing speed of 10 mm s^−1^. The printing bed was heated up to 45 °C to increase the drying step and help layer consolidation. The printer chamber humidity was kept above 60% to avoid nozzle blocking caused by paste drying.

### Post‐Processing of 3D Printed Carbon‐Based Electrodes

The as‐printed 3D electrodes were dried overnight at 60 °C and subsequently heat treated in a tubular furnace up to 800 °C at 1 °C min^−1^ under an inert N_2_ atmosphere (200 mL min^−1^). The resultant 3D electrodes were labeled as follows: 3D‐Gr, 3D‐Gr/CNT, 3D‐Gr/PF, and 3D‐Gr/oxPF.

### Membranes

Nafion212 (Dupont) membranes supplied by Sigma–Aldrich were soaked in Milli‐Q water (Millipore) at room temperature (20 °C) for 12 h before protonation in a 0.5 m H_2_SO_4_ solution for 48 h.^[^ ^76^ ^]^ Finally, they were rinsed and stored in Milli‐Q water for a minimum of 24 h until use.

### Custom‐Made Redox Flow Battery (RFB) Cell

Several preliminary prototypes of the Redox Flow Battery cell were designed using CAD Autodesk Inventor Professional 2015 and then built by 3D Prusa MK3S+ using PrusaSlicer in PLA and PP (Smart Materials). The final RFB cell was built in PTFE. The cell was sealed using Viton O‐rings and 5 mm thickness graphite plates current collectors. The cell components and assembly designs are shown in Figure 6 and available in the CSIC open‐access repository.^[^ ^72^ ^]^

### Physicochemical Characterization of the 3D Electrodes and Raw Materials

The yield of the raw materials after the thermal treatment at 800 °C was evaluated from the results obtained by TGA analysis (SDT600, TA Instruments). Experiments were performed up to 1000 °C at 10 °C min^−1^ under N_2_ flow (100 mL min^−1^). The electrical conductivity of the filaments conforming to each printed object after thermal treatment was measured by a 4‐point probe method applying a constant current between 2 and 30 mA using a VMP‐3e, Biologic potentiostat. The silver paste was applied to the filament cross‐section edges to minimize the resistance of contacts. The conductivity of the raw materials (Gr and GB‐derived carbon particles) was also measured following the 4‐point probe method using an SR‐4‐6l model from Everbeing International Co. For these measurements, it was necessary to prepare thin discs from pastes with the following formulation: 90 wt.% of the powdered (< 75 µm) and 10 wt.% of PTFE as a binder.^[^ ^61^ ^]^ For a given sample, at least 3 pellets were prepared and at least 6 measurements in each pellet were carried out to obtain its average conductivity value. Disc thickness was carefully controlled (120–180 µm) to minimize possible sample‐to‐sample deviations. SEM, (Quanta FEG 650) was used to characterize the morphology of the 3D electrodes (ETD detector and 25 kV). XRD measurements were carried out in a Bruker D8 Advance diffractometer, using a parallel beam (CuK_α_ at 40 mA and 40 kV) configuration. Measurement conditions were 0.008 °2θ step size and 1.2 s step time. Raman spectra were obtained in a Renishaw inVia Qontor equipped with a solid‐state visible laser at 532 nm, an 1800 lines mm^−1^ grating and a CCD deep depletion detector. An ultra‐long working distance objective was used to both focus and collect the laser beam on and from the sample, respectively. Typical measurement conditions were 1 accumulation of 10 s exposure from 3500–800 cm^−1^. BET‐specific surface areas were calculated from the N_2_ adsorption isotherms at −196 °C in an ASAP 2020 volumetric apparatus (Micromeritics).

### Mechanical Properties of the 3D Electrodes

Six cylinders (D: 10 mm and H: 10 mm) featuring 1 perimeter and 50% infill specimens (Figure S13a, Supporting Information) of each DIW 3D electrode composition, were printed and postprocessed similarly to the grid‐like electrode geometries. Uniaxial compressive strength was then measured in a universal testing machine (MTS, Sinergy USA 100N load cell) at 0.05 mm s^−1^.

### Electrochemical Characterization of the 3D Electrodes

All electrochemical measurements were conducted using an electrochemical workstation (VMP‐3e, BioLogic) at room temperature, using a 0.05 m VOSO_4_/1.0 m H_2_SO_4_ solution as electrolyte. Cyclic voltammetry (CV) and electrochemical impedance spectroscopy (EIS) experiments were performed in a three‐electrode cell using the 3D‐carbon‐based objects as working electrodes (WE). An Ag/AgCl/3.5 m KCl (0.205 V vs NHE) as the reference electrode (RE) and a graphite rod as a counter electrode (CE). All the potential values reported were referenced to the normal hydrogen electrode (NHE). Cyclic voltammetry (CV) experiments were carried out in a potential window of 0.7–1.4 V, at increasing scan rates (*v_scan_* = 20–100 mV s⁻¹) as the positive half‐cell. Similarly, for the negative half‐cell evaluation, experiments covered a potential window of −0.65 to 0.1 V, maintaining the same *v_scan_*. EIS measurements were performed at polarization potentials of 1.05 and −0.3 V for the positive and negative electrodes, respectively, applying a 10 mV AC amplitude over a frequency range from 10^6^ to 10^−1^ Hz.

Charge–discharge experiments were performed on the custom‐made redox flow battery (RFB) cell described above, holding as positive and negative electrodes the 3D objects under evaluation. All the tests were carried out by applying a current density of 15 mA cm⁻^2^, operating in a voltage range of 0.85–1.55 V, using a flow rate of electrolyte of 1 mL min⁻¹.

Pressure drop tests were carried out by coupling a U‐tube half‐full of water to the custom‐made RFB inlet/outlet. The pressure difference between the two points was calculated when running the cell with each different grid‐like electrode composition at different flow rates. Sessile drop wettability tests for 100% infill 10×10mm^2^ geometry specimens of DIW 3D electrodes (Figure S13b, Supporting Information) were carried out in a goniometer (DSA25S, KRUSS, Germany) with a 3 µL drop of the same electrolyte (0.5 m VOSO_4_ and 1 m H_2_SO_4_).

## Conflict of Interest

The authors declare no conflict of interest.

## Author Contributions

P.R.L. carried out most of the experiments, analyzed the results, and designed the RFB cell. A.C. and D.B. contributed to the electrochemical characterizations and analysis. M.A.M.‐M. measured the electrical conductivity of the raw materials and performed the Raman characterization of the materials. M.A.M.‐M. and J.A.M. supervised the project. V.G.R., C.B., R.S., and Z.G. conceived the idea, supervised and discussed the project, and achieved the funding acquisition. All authors discussed and commented on the manuscript.

## Supporting information



Supporting Information

Supplemental Video 1

Supplemental Video 2

Supplemental Video 3

Supplemental Video 4

## Data Availability

The data that support the findings of this study are available from the corresponding author upon reasonable request.
