# Supplementary material for: Direct Ink Writing of 3D‐Structured All‐Carbon Electrodes with High Electrical Conductivity for (Vanadium) Redox Flow Batteries
Source: Adv Sci (Weinh). 2025 May 15;12(22):2417641. doi: 10.1002/advs.202417641 (PMC12165035; doi:10.1002/advs.202417641)
Supplement: Supplementary file 1 — Supporting Information [file ADVS-12-2417641-s001.docx]

Supplementary Information

Direct Ink Writing of 3D-structured all-carbon electrodes with high electrical conductivity for (Vanadium) Redox Flow Batteries

Pablo Rodríguez Lagar, Alejandro Concheso, Daniel Barreda, Zoraida González, Miguel A. Montes-Morán, J. Angel Menéndez, Clara Blanco, Ricardo Santamaría, Victoria G. Rocha

Instituto de Ciencia y Tecnología del Carbono, INCAR-CSIC, Francisco Pintado Fe, 2, Oviedo 33011, Spain

E-mail: vgarciarocha@incar.csic.es

**Experimental**

Elemental analysis of carbon (C), hydrogen (H), nitrogen (N), and sulfur (S) was conducted using 1 mg of sample via complete combustion in a LECO CHNS-932 analyzer. Oxygen content was separately determined through pyrolysis using a LECO VTF-900.

1. **Table S**1. Raw materials characteristics

|  | **Elemental Analysis (wt.%)** | | | | |  |
| --- | --- | --- | --- | --- | --- | --- |
| Raw materials | C | H | N | S | O | PS^a)^  (μm) |
| **Gr** | 99.94 | 0 | 0 | 0 | 0.09 | <75 |
| **PF** | 95.17 | 0.10 | 4.77 | 0.49 | 0.14 | L^b)^:80  D^c)^:7 |
| **oxPF** | 94.05 | 0.08 | 4.78 | 0 | 0.47 | L^b)^:80  D^c)^:7 |
| **CNT** | 99.58 | 0 | 0 | 0 | 0.16 | L^b)^:5-9  D^c)^:0.11-0.15 |
| **GB** | 93.06 | 4.49 | 1.13 | 0 | 1.18 | <75 |
| **oxGF** | 99.7 | 0 | 0 | 0.01 | 0.14 | D^c)^:7 |

a) PS: Particle Size

b) L: length

c) D: diameter


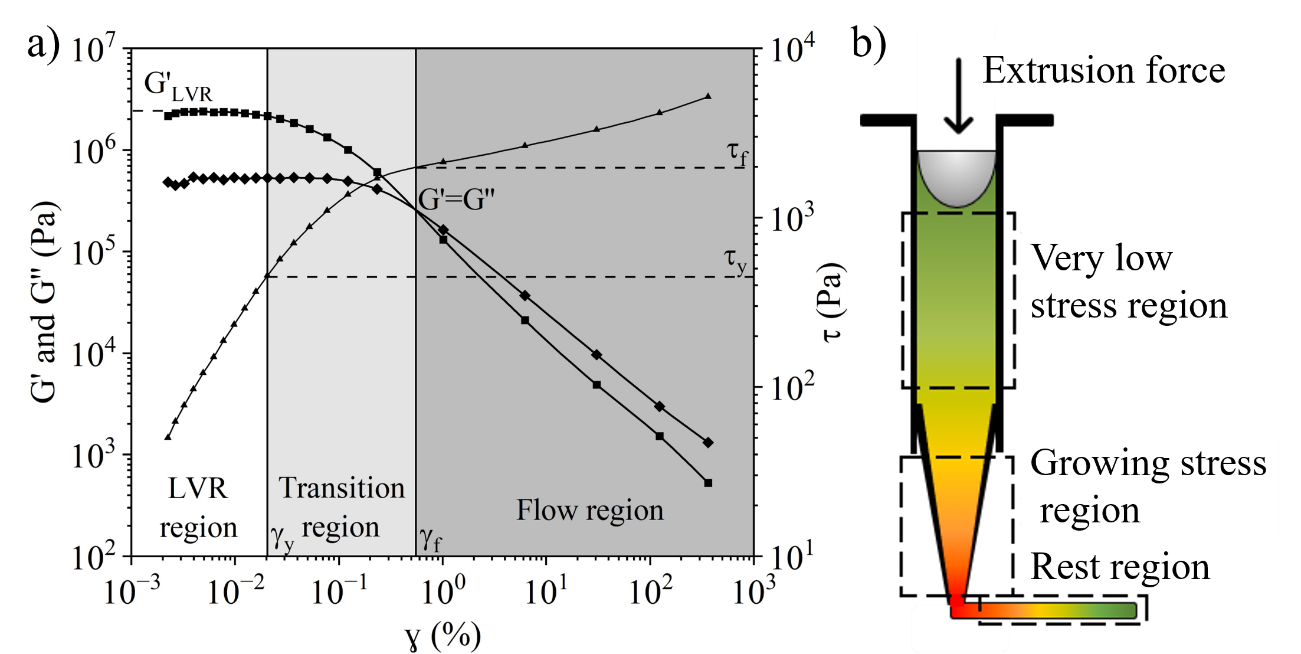


**Figure S1**. a) A typical amplitude sweep test of printable shear thinning pastes with the three well-defined rheological regions and transition values; b) stress profile experienced by the paste during the printing process.


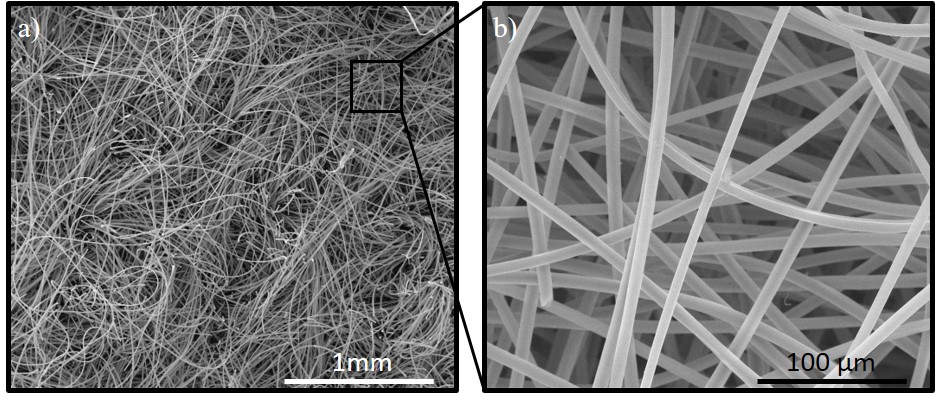


**Figure S2.** SEM images of the oxidized commercial graphite felt (oxGF) material used as benchmark for comparative purposes


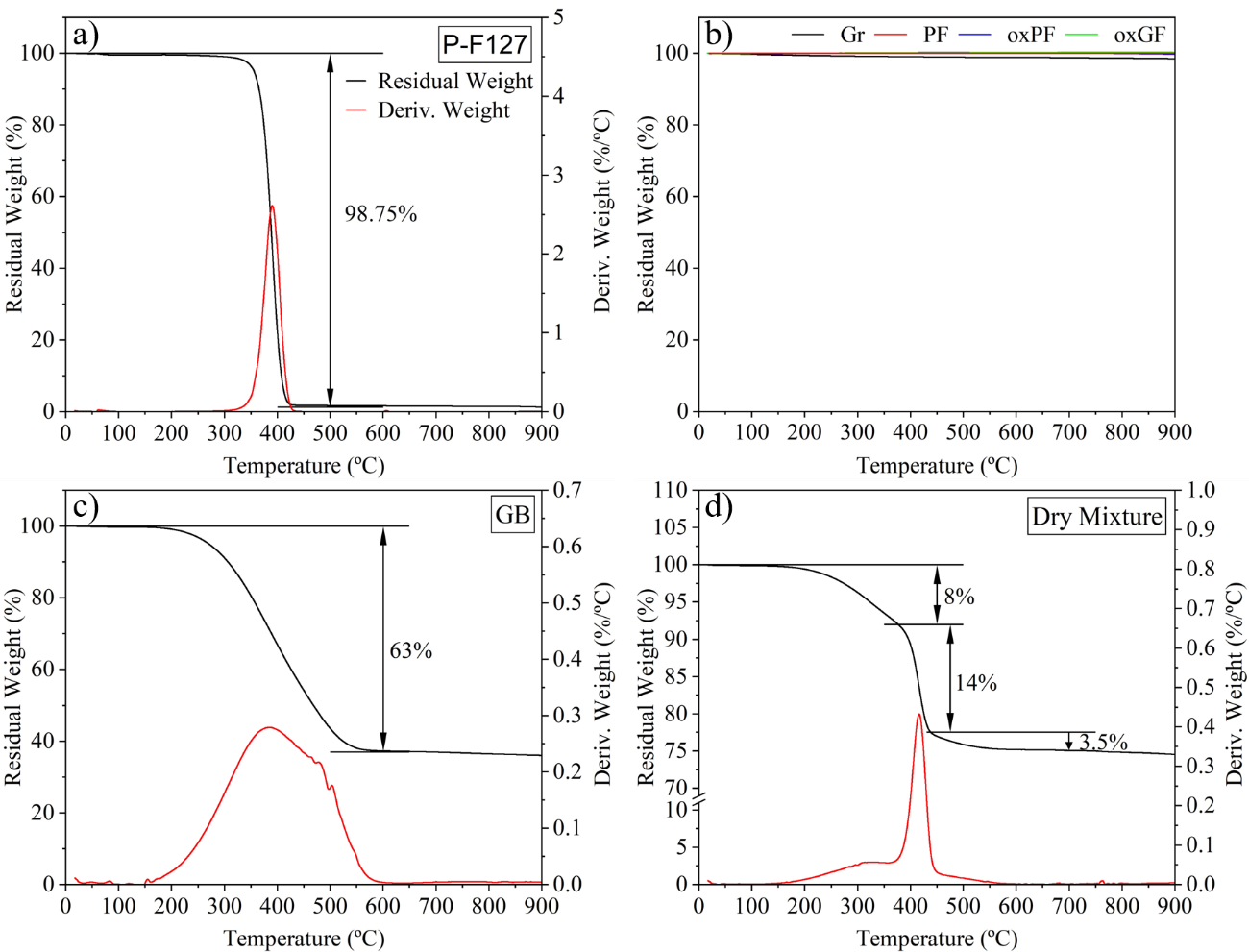


**Figure S3.** Thermogravimetric analysis under inert (N_2_) atmosphere of the different raw materials and dried pastes. The black lines correspond to the residual weight signal, and the red lines to the derivative of such signal with temperature.


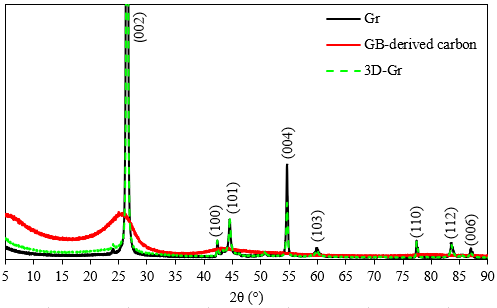


**Figure S4.** XRD profiles of the 3D-Gr electrode and its two components Gr and GB-derived carbon. The signal of the latter has been amplified for clarity in the comparison.

**

**

**Figure S5.** Raman spectra of the 3D-Gr electrode and its two components Gr and GB-derived carbon.


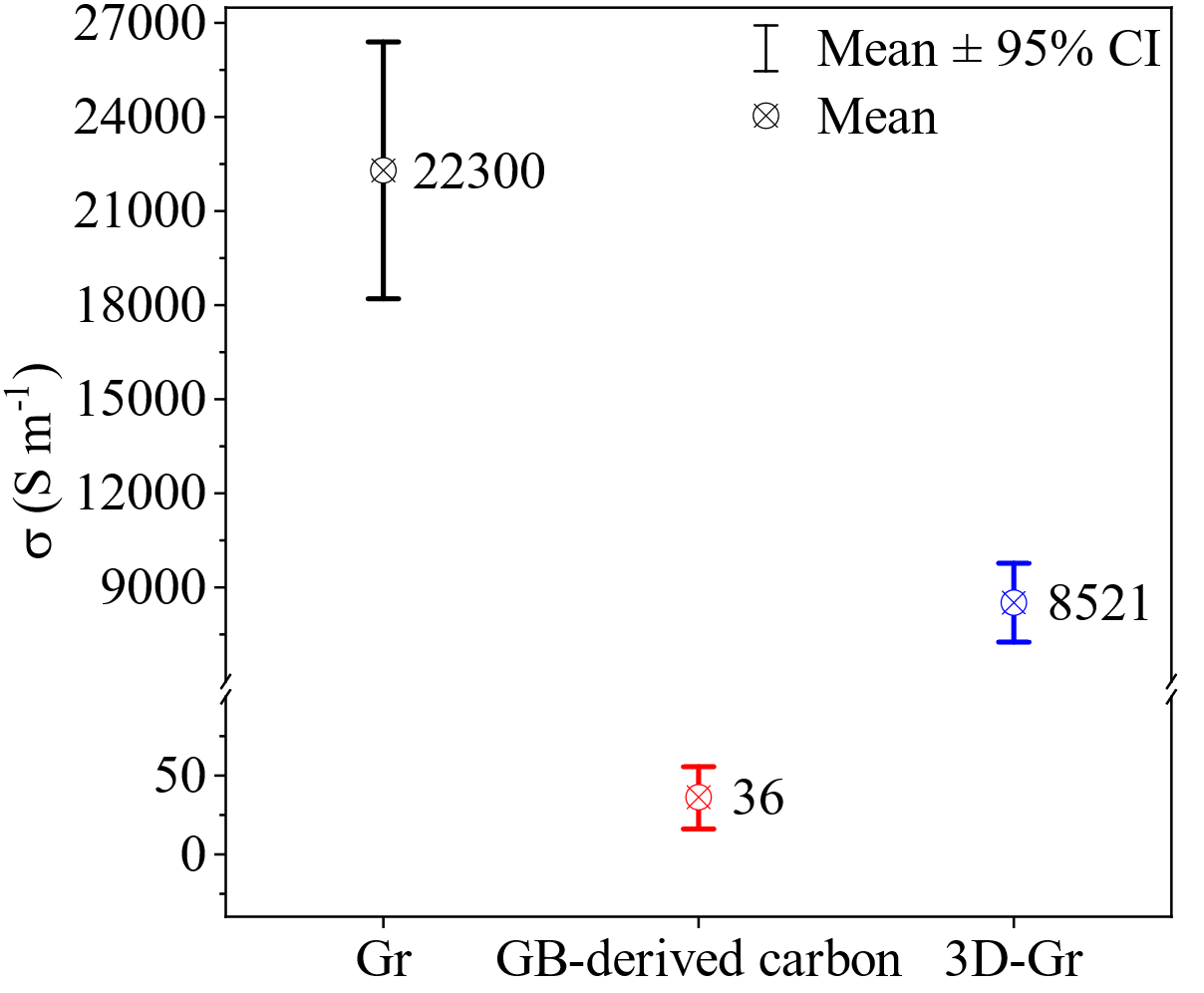


**Figure S6.** Electrical conductivity of the 3D-Gr electrode and its components, Gr and GB-derived carbon particles.

**Electrochemical characterization**

**Table S2.** Electrochemical data calculated for the positive and negative electrodes from the CVs recorded at 20 mV s^-1^

| Positive electrode | **3D-Gr** | **3D-Gr/CNT** | **3D-Gr/PF** | **3D-Gr/oxPF** | **oxGF** |
| --- | --- | --- | --- | --- | --- |
| **I_a_ (mA)** | 51.33 | 48.81 | 43.64 | 46.41 | 153.7 |
| **I_c_ (mA)** | 51.25 | 49.96 | 44.7 | 47.12 | 160.3 |
| **I_a_/I_c_** | 1.00 | 0.98 | 0.98 | 0.98 | 0.96 |
| **ΔE (mV)** | 301 | 180 | 279 | 323 | 369 |

| Negative electrode | **3D-Gr** | **3D-Gr/CNT** | **3D-Gr/PF** | **3D-Gr/oxPF** | **oxGF** |
| --- | --- | --- | --- | --- | --- |
| **I_a_ (mA)** | 46.08 | 31.38 | 34.77 | 39.61 | 288.22 |
| **I_c_ (mA)** | 51.2 | 46.16 | 57.57 | 65.74 | 290.70 |
| **I_a_/I_c_** | 0.9 | 0.68 | 0.60 | 0.60 | 0.99 |
| **ΔE (mV)** | 301 | 186 | 371 | 380 | 339 |

**
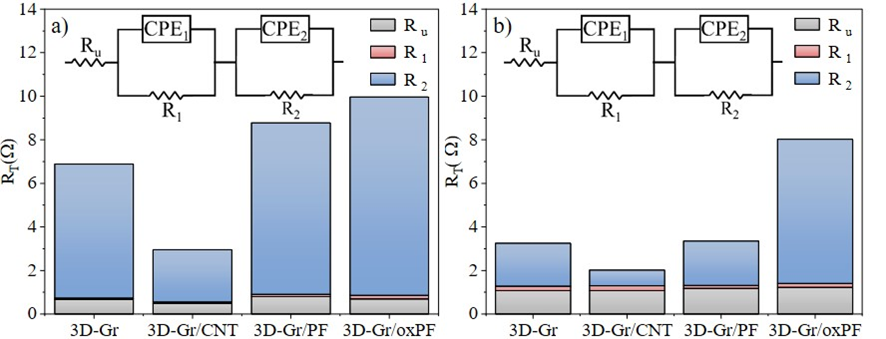
**

**Figure S7.** Equivalent circuits and resistances values from Nyquist plots from EIS experiments in three-electrode half-cell. a) 3D electrodes as positive half-cell b) 3D electrodes as negative half-cell

A flow-through three-electrode half-cell design (Figure S5) was used to investigate the electrochemical response of the different 3D carbon-based electrodes, under vanadium electrolyte flow rates ranging from 2 to 38 mL∙min⁻¹ within the same potential window. The Electrochemically Active Surface Area (ECSA) values were estimated using double-layer capacitance (Cdl) measured in a non-faradaic potential (0.35-0.64 V vs NHE) window and using a general specific capacitance of 5 µF∙cm⁻² in KOH 6 M^[1]^.

CE

REF

WE

VO^2+^

VO^2+^

**Figure S8.** Experimental setup of the flow-through three-electrode half-cell configuration used.

Linear sweep voltammetry (LSVs) experiments, at different flow rates, were performed in this experimental setup using grid like geometries of 3D-Gr and 3D-Gr/CNT as positive half-cell working electrodes. It is expected that this cell configuration behaves closer to a real flow cell, with little or no diffusional restrictions, and the results obtained should be more helpful to understand and select the best electrode materials. For instance, Figure S6 shows the results obtained within this cell and there are significant differences in the oxidation overpotentials and anodic currents measured during the charge step, being much lower for the formulation containing the CNT as it was already observed for the static CV experiments (Figure 5a). It is remarkable that in this experiment the highest intensity was measured on the 3D-Gr/CNT electrode and not on the 3D-Gr, contrary to the results shown in Table S2 (derived from similar experiments but performed using a conventional 3-electrode cell). That results demonstrate that the CNT decorating the electrode surface are very active favoring the chemical reactions.


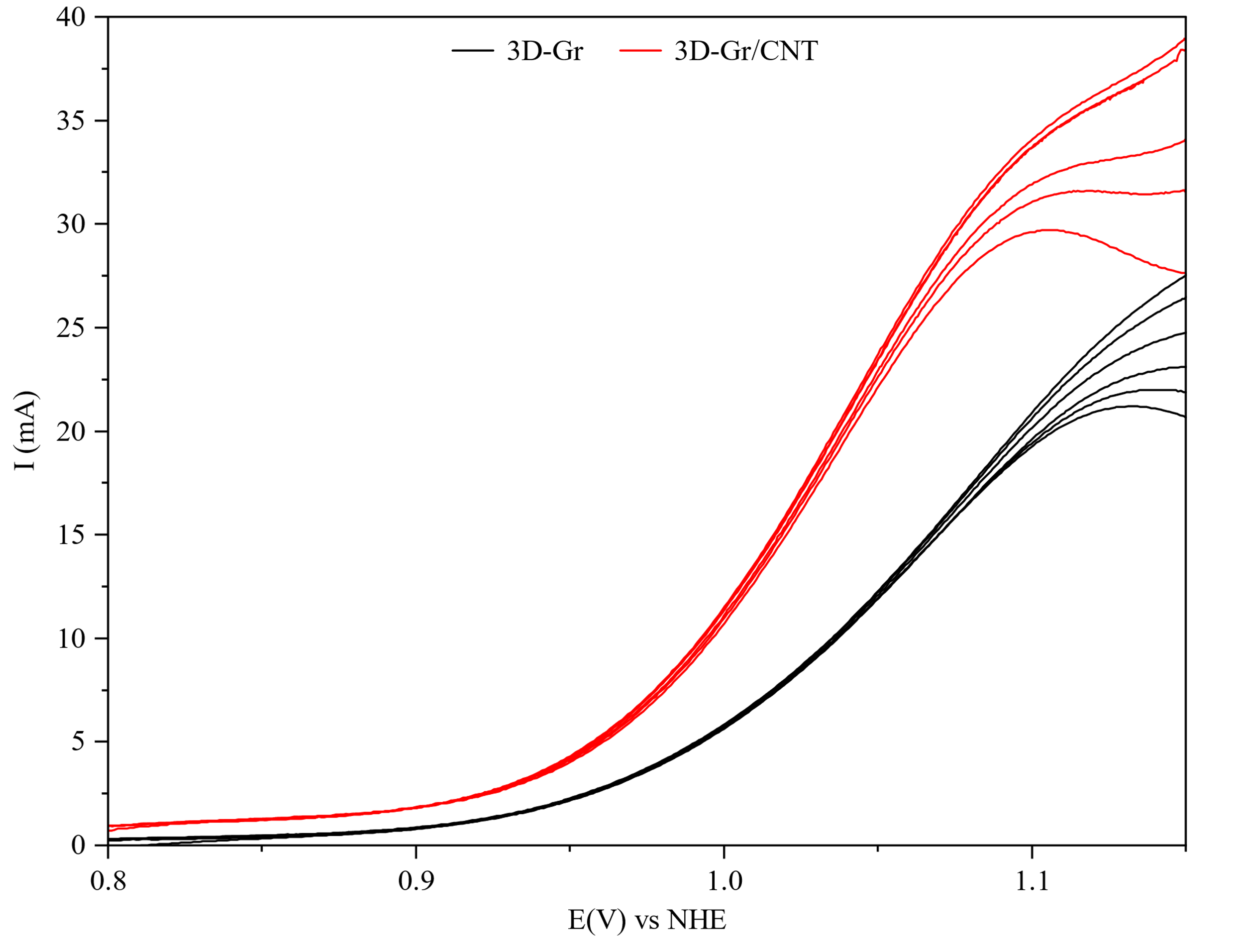


**Figure S9.** a) LSVs recorded at 1 mV s −1 at different flow rates ranging from 2 to 38 mL min^-1^ in 0.05 M VOSO_4_ / 1.0 M H_2_SO_4_ solution for the positive reaction at the VRBF.

In addition, at low potentials (Figure S9) small differences can be observed in the capacitive current in the potential range where no faradaic reactions are developed. These residual currents must be related to differences of specific surface area between the electrodes. BET specific surface areas determined by N_2_ adsorption at cryogenic were in all cases < 10 m^2^g^-1^. On the other hand, the calculated ECSA was slightly different for these two electrodes (140 cm^2^ for 3D-Gr vs. 179 cm^2^ for 3D-Gr/CNT). In order to get a deeper knowledge on the electrochemical system some experiments were also performed using a rotating disk electrode (RDE), thus avoiding diffusional limitations and, at the same time, allowing the acquisition of quantitative results from the Koutechy-Levich equations. In order to perform these RDE experiments new electrodes geometries where selected and 3D printed (using the same Direct Inkjet Writing methodology, Figure S10). Finally, they were fit in a custom-made holder for their subsequent coupling to the RDE instrument.


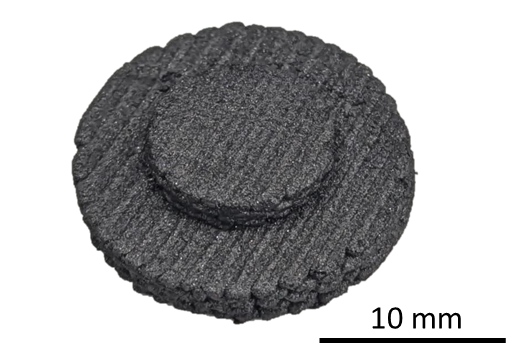


**Figure S10.** Photograph of the 3D-Gr electrode for its electrochemical characterization in an RDE. Electrodes with 100% infill geometry are required for these experiments.

Aiming to estimate/calculate the kinetic parameters related to the 3D-Gr and 3D-Gr/CNT electrodes, LSV experiments were performed on a RDE, varying the rotation rate from 500 to 3000 rpm and using a potential window between 0.8 – 1.15 V (vs. NHE) at scan rate of 1 mV s^-1^(Figure S11 and Table S3).


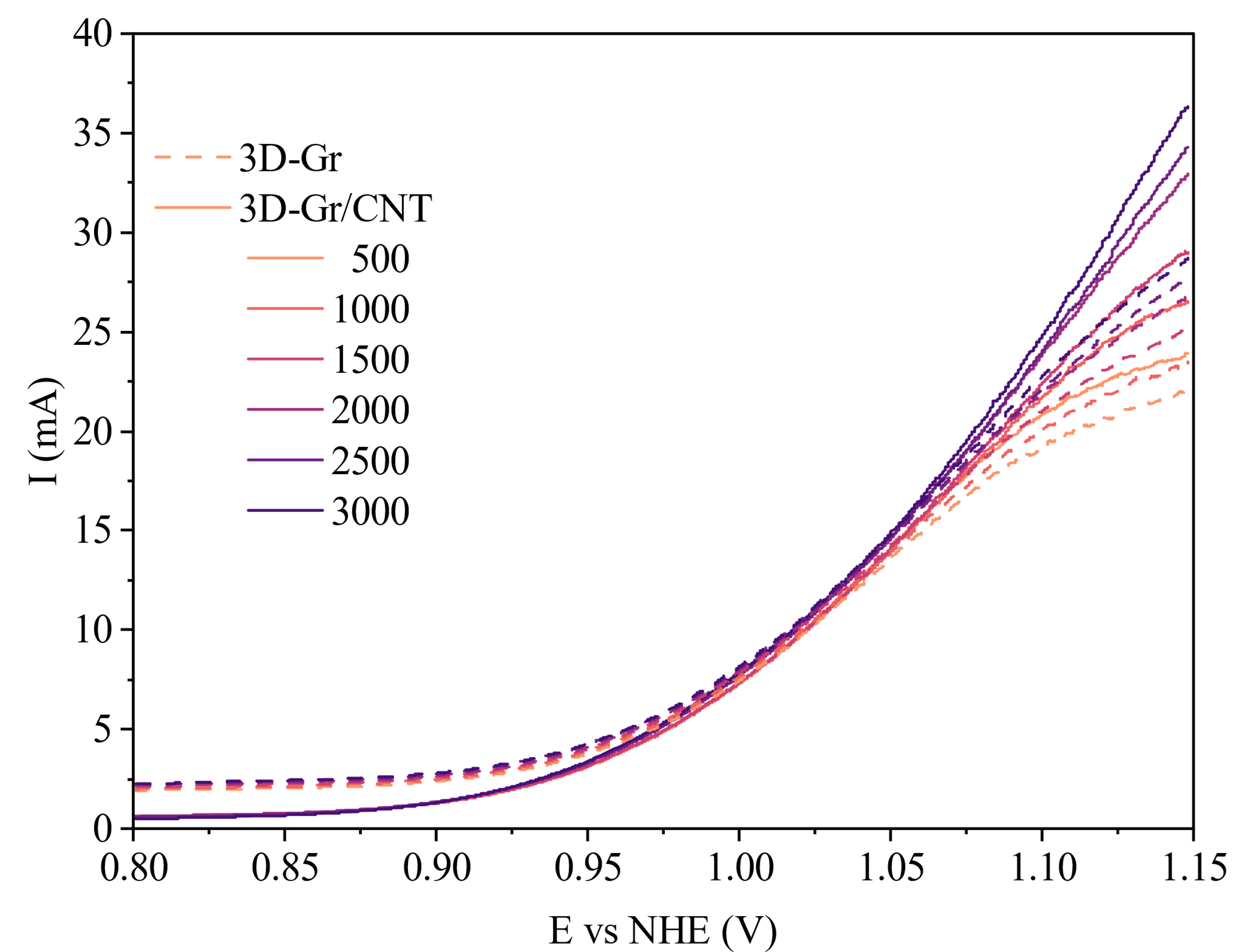


**Figure S11**. LSV recorded at 1 mV s^−1^ at different RDE rotation speeds in 0.05M VOSO_4_ / 1.0 M H_2_SO_4_ solution.

**Table S3.** Kinetic parameters derived from the LSV measurements performed on the RDE using different composition of 3D carbon based working electrodes with 100% infill geometry.

|  | **3D-Gr/CNT** | **3D-Gr** |
| --- | --- | --- |
| **K (cm/s)** | 2.81x10^-3^ | 2.32x10^-3^ |
| **α** | 0.263 | 0.317 |

Although the results obtained on the RDE reinforce our hypothesis that the addition of CNT can boost redox reactions rate as reflected in a 20% increase in the value of Ka, it is worth considering that these calculations can be questioned. One of the main assumptions for such a kinetic analysis, see ^[2]^for details, is that the mass transport to each point of the catalyst surface is identical, and that might be approximately true for a flat surface (a metal surface, for example) but some deviation might be introduced when rough surfaces (Figure 3) are used, and when nanoparticles are distributed on the surface. ^[3,4]^ Therefore, the qualitative results shown in Figure S9 are a very clear indication of the differences found between the two electrodes although quantitative results cannot be obtained as there is no mathematical model that can be easily applied to this system


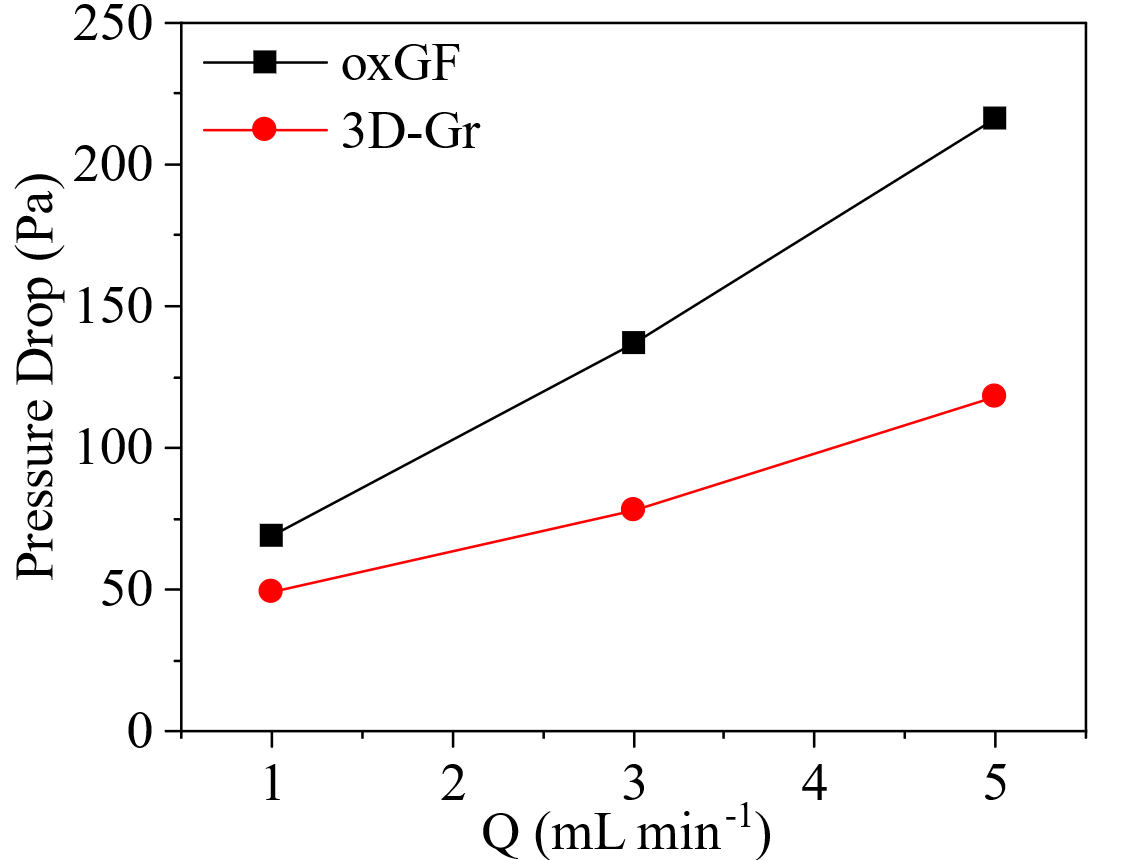


**Figure S12.** Pressure drop at different flow rates measured in the custom-made RFB cell for the benchmark material (oxidized graphite felt (oxGF)) and the grid like geometry of 3D-Gr composition

**
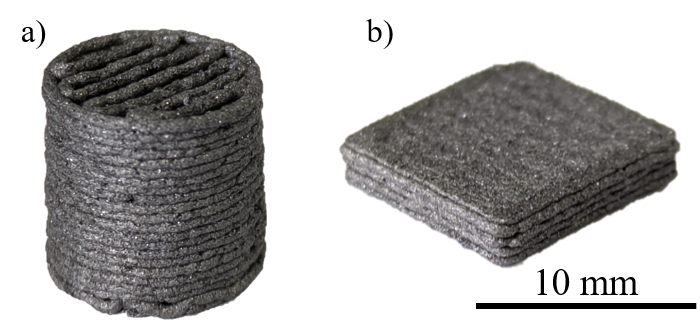
**

**Figure S13.** Photographs showing different geometries of 3D-Gr composition a) cylindrical specimen D:10 mm, H:10 mm 1 perimeter and 50% infill geometry for uniaxial compression strength measurements and b) 10x10 mm^2^ 100% infill square geometry used in the wettability test.


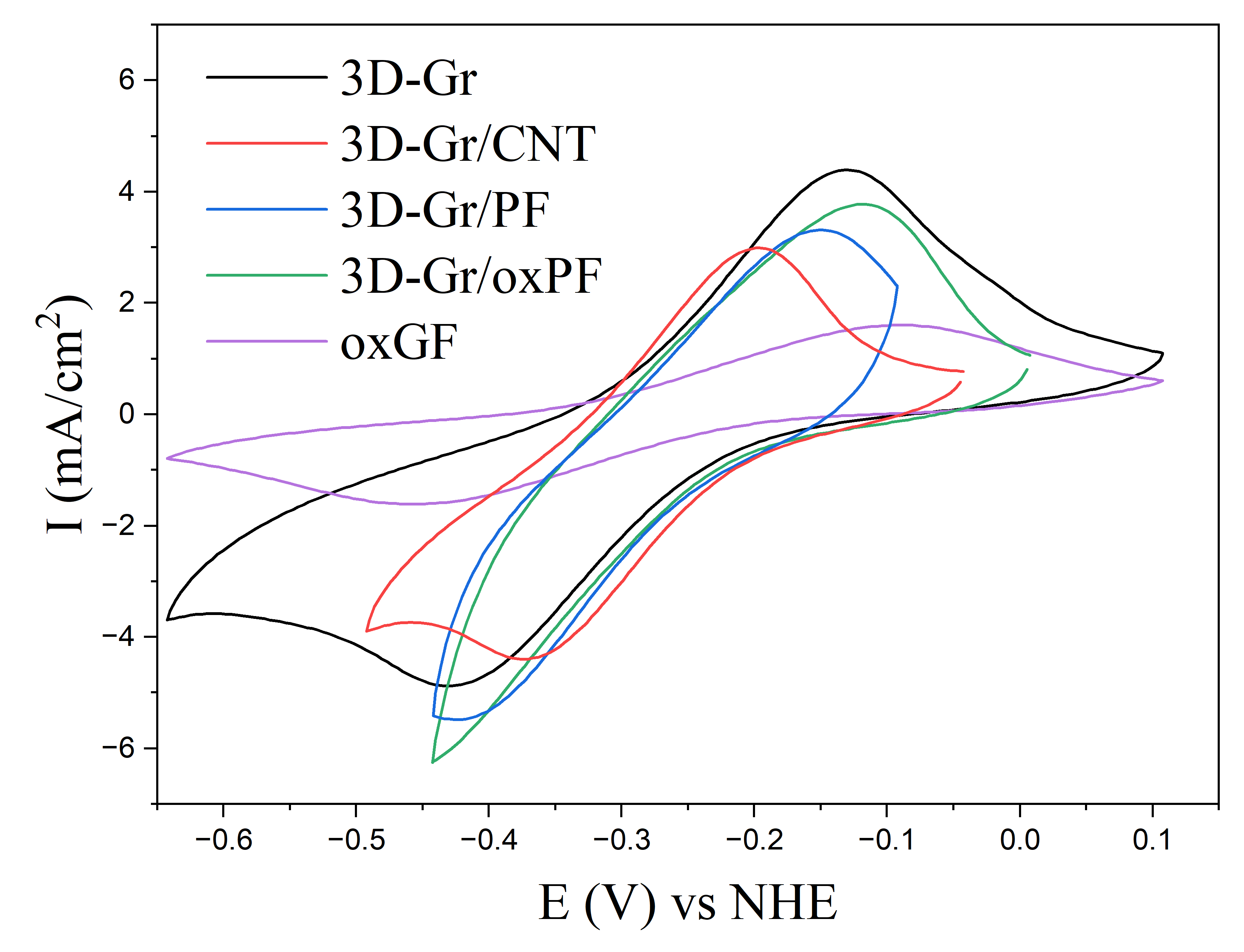

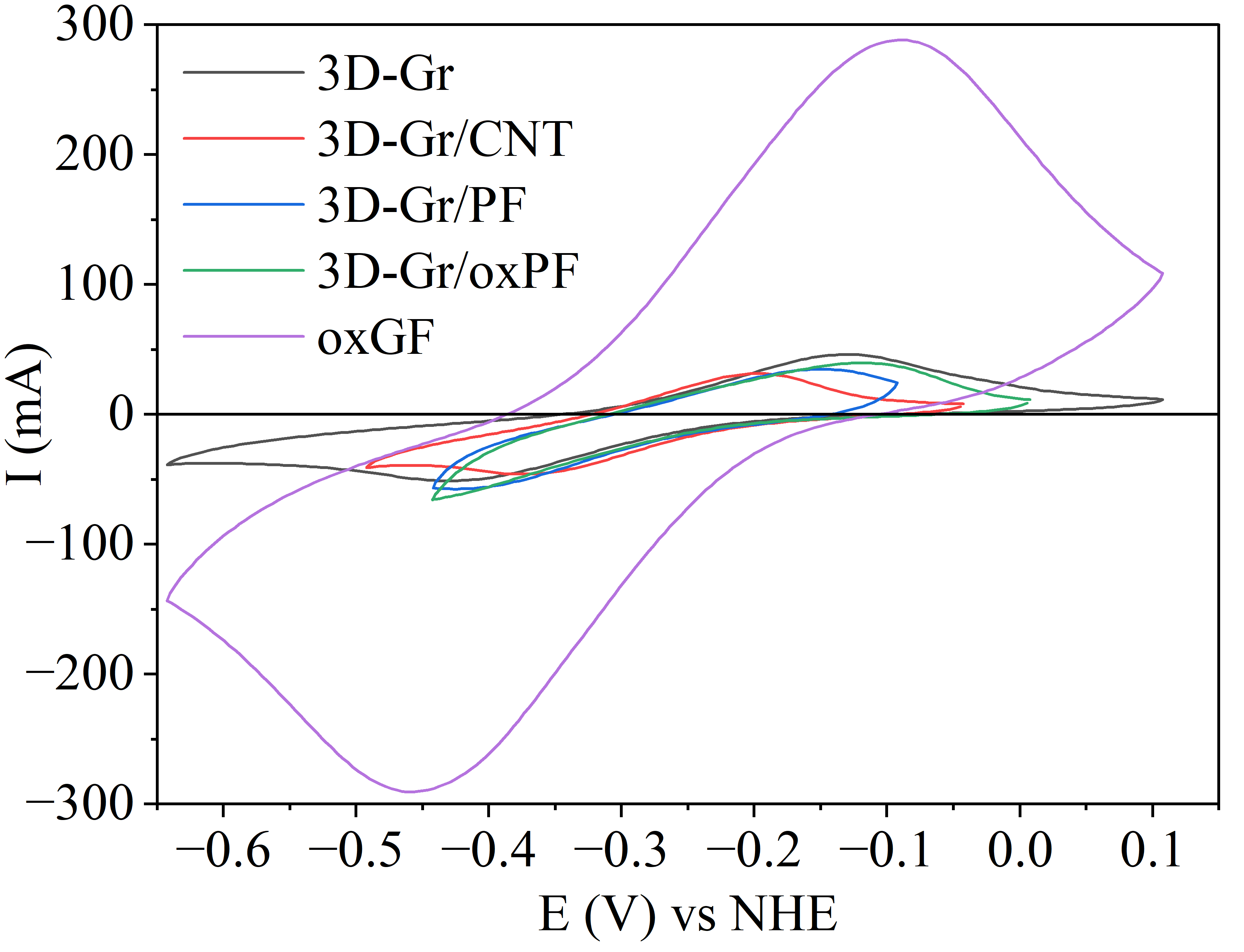


**Figure S14.** CVs recorded at 20 mVs^-1^ on the different electrodes in the positive potential window: a) (Figure 5a); b) surface specific current intensities, using 10.5 cm^2^ and 180 cm^2^ of external surface areas for all 3D electrodes and oxGF, respectively.

**References**

[1] H. Ji, X. Zhao, Z. Qiao, J. Jung, Y. Zhu, Y. Lu, L. L. Zhang, A. H. MacDonald, R. S. Ruoff, *Nat Commun* **2014**, *5*.

[2] Allen J. Bard and Larry R. Faulkner, Electrochemical Methods: Fundamentals and Applications, New York: Wiley, 2001, 2nd ed., *Russian Journal of Electrochemistry* **2002**, *38*, 1364.

[3] M. Inaba, A. W. Jensen, G. W. Sievers, M. Escudero-Escribano, A. Zana, M. Arenz, *Energy Environ Sci* **2018**, *11*, 988.

[4] M. Inaba, J. Quinson, M. Arenz, *J Power Sources* **2017**, *353*, 19.
